# Carp edema virus surveillance in the koi trade: early detection through shipping environment sampling and longitudinal monitoring of CEV outbreaks in a wholesaler facility

**DOI:** 10.1186/s13567-025-01476-1

**Published:** 2025-03-04

**Authors:** Laetitia Montacq, Marine Baud, Hélène Giummarra, Doriana Flores, Laurane Pallandre, Cécile Caubet, Sokunthea Top, Timothée Vergne, Laurent Bigarré, Stéphane Bertagnoli

**Affiliations:** 1 IHAP, Université de Toulouse, INRAE, ENVT, 31300 Toulouse, France; 2 ANSES, Laboratoire de Ploufragan-Plouzané-Niort, 29280 Plouzané, France; 3 NeoVirTech SAS, 31100 Toulouse, France

**Keywords:** Carp, *Cyprinus carpio*, transboundary animal disease, *Poxviridae*, genotyping

## Abstract

Carp edema virus (CEV), a member of the *Poxviridae* family, has been a significant pathogen in koi and common carp since its initial identification in Japan during the 1970s. CEV, the causative agent of Koi Sleepy Disease (KSD), can cause high mortality rates and has been reported in many countries and is often linked to the fish trade. The virus is typically detected through DNA analysis of gill tissues, where the highest viral loads are found. However, traditional sampling methods, such as gill sampling, are lethal, complicating routine surveillance, particularly in asymptomatic or high-value koi. This study aimed to evaluate nonlethal sampling methods for CEV surveillance in the koi trade. We analysed various shipping environment samples, such as shipping water and fish bag swabs, alongside gill swabs from anaesthetised fish and gills from naturally deceased fish. Using qPCR, we found that the sensitivity of environmental samples, particularly shipping water, was greater than that of direct fish samples. Latent class modelling estimated that the sensitivity associated with 1.5 mL shipping water samples was greater than 89%, making them a reliable alternative for early detection. All detected variants belonged to genogroup II. Some post-import outbreaks shared variants with earlier outbreaks or shipping environment samples, suggesting that the detected DNA generally reflected infectious particles rather than just free environmental DNA and indicating that CEV can go unnoticed for several months after importation. These findings highlight the utility of environmental samples for effective, non-invasive surveillance and improved biosecurity management in the koi trade.

**Supplementary Information:**

The online version contains supplementary material available at 10.1186/s13567-025-01476-1.

## Introduction

Carp edema virus (CEV), which belongs to the *Poxviridae* family, was first identified in the 1970s in Japan as the agent responsible for Koi sleepy disease (KSD), which causes mass mortalities of juvenile koi on farms across several prefectures [1]. Since then, the disease has emerged in numerous countries, affecting both koi and common carp [2–4]. Fish infected with CEV typically suffer from an acute form of the disease, showing nonspecific clinical signs such as apathy, anorexia, generalised oedema, skin and gill lesions, and high mortality rates of up to 70–100% within 2 weeks [2, 5–7].

CEV has a genome consisting of double-stranded DNA that is approximately 460 kbp in size and contains 392 predicted genes [8, 9]. Until the recent publication of a unique complete genome assembly [9], only two partial sequences of the *P4a-* and *P4b*-encoding genes were available [8]. Before 2021, phylogenetic analyses were performed on two short fragments (< 600 bp) of the *P4a* coding gene. On the basis of these analyses, all the samples analysed at that time clustered into two genogroups (gI and gII), which differed in terms of substitutions [10–12]. More recently, phylogenetic analyses were performed on a larger fragment (approximately 900 bp) of the *P4a* gene and on two other CEV genes, namely, DNA binding viral core protein 8 (VP8) and uracil DNA glycosylase (UDGS), which support the same clustering into gI and gII [13, 14]. Genogroup I is typically associated with common carp, which are usually descendants of the European subspecies *Cyprinus carpio carpio*. On the other hand, genogroup II is associated mainly with koi, which are coloured variants selected primarily from Asian carps, usually referred to as *Cyprinus carpio haematopterus*, *Cyprinus carpio rubrofuscus*, and/or *Cyprinus rubrofuscus*, among others [11, 12, 15–22].

Oyamatsu et al*.* published the first molecular diagnostic tool for detecting CEV in 1997 [23]. This method consists of nested PCR targeting a portion of the *P4a* coding sequence (erroneously known as the 5’ UTR at that time). Since then, new conventional PCR and real-time PCR methods (TaqMan^®^ and SYBR^®^ green-based) have been developed to target the *P4a* gene [11, 24, 25]. Studies have also investigated the distribution of the CEV in carp bodies and reported that gills presented the highest DNA loads, which was consistent with the histological and electron microscopic findings. Therefore, detection is typically performed subsequent to DNA extraction from the gills of deceased fish [1, 7, 24].

Most outbreaks of KSD occur after stressful events, such as water temperature variations and transportation, such as commercial exchanges or restocking procedures [3]. These outbreaks can be managed by supportive treatment, such as prolonged salt baths (0.5% NaCl). This inexpensive treatment effectively reduces both symptoms and mortality rates, making it widely used by koi farmers [7, 26]. However, this procedure appears to be ineffective at preventing the spread of CEV globally, as healthy carriers can still transmit the virus horizontally. European laboratories have already detected CEV in koi from recent imports from countries such as Israel and Japan [2, 24, 27, 28]. Additionally, phylogenetic analyses based on the *P4a* gene suggest that CEV spreads through multiple imports of infected common carp and koi carp [4, 13].

To prevent the dissemination of CEV between regions, particularly via the international trade of koi, testing the presence of the virus in transport bags before the introduction of the fish and without lethal sampling would be useful. Indeed, lethal sampling is often unacceptable for systematic surveillance, especially in apparently healthy and/or expensive fish. Additionally, in the context of the surveillance of another carp virus with gill tropism (i.e., *Cyvirus cyprinidallo 3,* also known as cyprinid herpesvirus 3), Bergmann et al*.* demonstrated that the sensitivities of gill swabbing and gill sampling (after euthanasia) were not different [29]. We therefore questioned whether shipping water or gill swabs (performed under anaesthesia) could serve as an alternative to tissue sampling for CEV surveillance in the koi trade. Since CEV is not cultivable in vitro [8, 30–33], we monitored mortality events both near and long after importation (from days to months) to check if the same variants were circulating, which would suggest that the DNA detected in imports reflected the presence of infectious particles rather than just free environmental DNA.

## Materials and methods

### Study site and sample collection

All samples were collected from a French ornamental fish wholesaler facility where CEV had previously been detected and associated with fish losses. Some koi were bred locally, whereas others were imported from Japan. Typically, CEV outbreaks in this facility occurred after importation. Therefore, we sampled each koi import between 2019 and 2022 (a total of 3 shipments).

The fish consignments were obtained through a Japanese forwarding agent, and the shipment took approximately 72 h. Whether any chemical treatment was applied to the shipping water to prevent transport-stress mortality was unknown. During 2019, 2020 and 2022 shipments, 21, 20 and 22 bags, respectively, were imported (60–150 fish per bag, 7–18 cm in length). Upon arrival, the fish were transferred to 500-L aquariums with a ratio of one bag per aquarium, except for one 2022 bag, which was split into two batches (22-F1 and 22-F2). Batch identification labels include a two-digit year, a letter denoting the breeder, and a number to differentiate batches from the same breeder if needed. For example, *19-A1* stands for the first batch from farmer A in 2019. Day 0 refers to the day the fish arrived, including shipping environment samples and any fish that died during transportation, while subsequent days are labelled day 1 for the day after import, day 2 for the day after, and so on.

For the 2019 shipment, the fish did not receive a bath treatment on arrival, and water renewal procedures commenced. Two days later, the renewal process was halted, and the salinity was artificially raised to 3 g of NaCl per litre for most batches. After four additional days, the salinity level was either increased to 6 g/L for most batches or maintained at 3 g/L. For both the 2020 and 2022 shipments, all batches were treated with saltwater containing 6 g/L NaCl (final concentration) and methylene blue (at unknown concentrations) upon arrival. No water renewal occurred during the first few days following arrival.

Upon opening each bag, approximately 20 mL of shipping water was collected before the fish were transferred to their aquaria without shipping water. For the 2019 and 2022 shipments, dry swabs were rotated against the inside of the recently emptied bags, and the swab tips were cut off and placed in empty 1.5 mL Eppendorf^®^ tubes. Additionally, for the 2019 and 2022 shipments, the farm veterinarians collected gill swabs from five anaesthetised fish per batch four days and one day after arrival, respectively. Anaesthesia was performed individually using 2-phenoxyethanol in a bath (exact dose not indicated by the veterinarian). Gill swabbing ceased in 2020 due to restrictions imposed by the COVID-19 pandemic. The swabs tips were cut off and placed into 1.5 mL Eppendorf^®^ tubes. Water and swab samples were stored at −20 °C for future analysis.

Any fish that died during transport were collected and preserved at −20 °C. Since no fish died during the transport of the 2019 and 2022 shipments, those that died within five days of arrival were frozen at −20 °C. Moreover, any fish that died outside the immediate post-arrival period (i.e., the first five days) but during major mortality events were also collected and frozen at −20 °C.

### DNA extraction

#### Swabs: shipping bags and gills

To each tube containing gills or bag swabs, 500 µL of phosphate-buffered saline (PBS) was added, and the mixture was vortexed for 30 s. For bag swabs, 150–200 µL of the resulting suspension was used for DNA extraction, whereas for gill swabs, 5 volumes of 40 µL of the suspension were pooled per batch. The manufacturers’ instructions were followed to use the NucleoSpin^®^ RNA virus mini kit (Macherey–Nagel, Germany) or ONE-4-ALL genomic DNA minipreps (Bio Basic Inc., Canada), with proteinase K treatment and without an RNA carrier. The DNA was eluted into 50 µL of the provided elution buffer.

#### Water

##### 2019 shipment

For the 2019 shipment, 200 µL of water was extracted from approximately 20 mL of collected water with ONE-4-ALL genomic DNA minipreps (Bio Basic Inc., Canada) according to the manufacturer's instructions. For CEV-negative samples, the remaining 20 mL of shipping water was centrifuged for 4 min at 2000 × *g*; the pellet was then resuspended in the supernatant to a final volume of 200 µL and extracted using the same kit.

##### 2020 and 2022 shipments

For the 2020 and 2022 shipments, modifications to the lysis and membrane adsorption steps were made for DNA extraction from water to account for larger volumes. A total of 15 µL of 1 M TrisHCl, 79 µL of 0.5 M EDTA, 75 µL of 10% sodium dodecyl sulfate (SDS) in double-distilled water, and 200 µg of proteinase K (Macherey–Nagel, Germany) were added to 1.5 mL of thawed shipping water. The samples were incubated for 30 min at 55 °C. An equal amount of absolute ethanol (1.5 mL) was then added, and the NucleoSpin^®^ RNA Virus Mini Kit columns (Macherey–Nagel, Germany) were loaded with 650 µL of the mixture in succession (removing the flow-through) until the entire sample was processed. Washing and elution steps were performed following the manufacturer's instructions. The DNA was then eluted in 50 µL of the provided elution buffer.

#### Deceased fish

##### 2019 shipment and any fish that died during a major mortality event before the 2020 import

For the 2019 shipment, two gill arches were thawed and finely ground using a mortar and pestle with sterile sand and 2 mL of cell culture medium (Gibco^™^—OptiMEM^™^) containing penicillin, streptomycin and amphotericin B. The homogenised gill was then transferred to Eppendorf^®^ tubes and centrifuged for 10 min at 800 × *g*. A total of 200 µL of the supernatant was used for DNA extraction with ONE-4-ALL genomic DNA minipreps (Bio Basic Inc., Canada), following the manufacturer’s instructions. The DNA was eluted in 50 µL of the provided elution buffer.

##### 2020 and 2022 shipments and any fish that died during a major mortality event after the 2020 import

After thawing, the gills of deceased fish were collected. A small amount (approximately 200–300 mg) was diced and dissolved in a non-commercial lysis buffer consisting of 900 µL of Tris–EDTA, 50 µL of 0.5 M EDTA, 50 µL of 10% SDS, and 200 µg of proteinase K (Macherey–Nagel, Germany). The samples were incubated at 55 °C for a minimum of 30 min until the gills were visibly lysed, leaving only the cartilaginous components. Each tube was then supplemented with 430 µL of an aqueous solution saturated with sodium chloride. The samples were subsequently centrifuged at 10 000 × *g* for 30 min at 4 °C. The resulting supernatant was transferred into a fresh tube containing 3 mL of absolute ethanol. The NucleoSpin^®^ RNA virus mini kit spin column (Macherey–Nagel, Germany) was loaded successively with 650 µL. Washing and elution steps were performed following the manufacturer’s instructions. The DNA was eluted with 50 µL of the provided elution buffer.

### Detection of CEV DNA

DNA concentration was measured via spectrophotometry with the CLARIOSTAR Plus system (BMG Labtech). To detect and quantify CEV DNA, we used quantitative PCR (SYBR^®^ Green) as described by Adamek et al. [25]. For quantification, we used a recombinant plasmid containing a portion of the *P4a* gene diluted from 10^1^ to 10^7^ copies. Extracts with high DNA concentrations were suitably diluted (tenfold or 100-fold) to prevent saturation of the SYBR^®^ Green signal. The detailed qPCR protocols are available in Additional file 1. Depending on the sampling method, the qPCR results are expressed either as the number of genome copies per volume of DNA extracted (comparison between shipping water and fish bag swabs), the number of genome copies per quantity of DNA extracted (gills and gill swabs), or the number of genome copies per volume of water extracted (shipping water).

The samples were considered positive if the following criteria were met: (1) the Ct value was less than 40 and (2) the two melting curves were superimposed on each other and on the plasmid range melting curves. Otherwise, positivity was confirmed by migrating the qPCR product on an agarose gel or performing a conventional PCR targeting *P4a* followed by Sanger sequencing, or both. If the automatic qPCR quantification fell below the plasmid range (i.e., < 10^1^ copies/2 µL) but the sample was positive, the qPCR result was not quantifiable but was arbitrarily displayed at 5 copies/2 µL of tested extract. If the DNA extract was diluted tenfold before qPCR, then the result was displayed at 50 copies/2 µL of undiluted extract.

To assess the amplifiability of DNA in fish samples, specifically from gill and gill swabs, we used the TaqMan qPCR method outlined by Gilad et al*.* [34], which targets the carp glucokinase gene.

### Sanger sequencing and phylogenetic analyses

#### PCR and Sanger sequencing

Conventional PCR was utilised for amplicon production, employing various protocols and primers on the basis of the sequencing date and team responsible (see Table 1 and Additional file 1 for detailed protocols). Typically, Sanger sequencing is followed by conventional PCR amplification. If only one double peak was observed in the entire sequence, the sample was divided into two sequences, for example, *2019_day00_A2_shipping_water 1/2* and *2019_day00_A2_shipping_water 2/2*. For the most recent samples and those that exhibited ambiguous chromatograms (i.e.*,* more than one double peak), Sanger sequencing was performed on several clones after initially cloning the PCR product. For samples that were cloned before sequencing, fraction tags (e.g., 1/3, 2/3, and 3/3) were used to show different sequences if needed.

**Table 1:** **PCR primers utilised in this study for CEV detection and Sanger sequencing**

PCR type		Primer name	Sequence (5’–3’)	Use	References
qPCR		CEV_TiHo_qF	CAT TTC CTA GTT TGT ATG GCA AG	Detection and quantification of CEV DNA in all samples	[25]
		CEV_TiHo_qR	TGA TGA TTG GAA TAA GAT GTC TGT C		
Nested PCR	First round	CEV_for_B	ATG GAG TAT CCA AAG TAC TTA G	Amplicon production for Sanger sequencing of 2019 and February 2020 samples. Second round was performed only if first round amplification was weak	[15]
		CEV_rev_J	CTC TTC ACT ATT GTG ACT TTG		
	Second round	CEV_for_B_int	GTT ATC AAT GAA ATT TGT GTA TTG		
		CEV_rev_J_int	TAG CAA AGT ACT ACC TCA TCC		
Semi-nested PCR (used with CEV_rev_J & CEV_rev_J_int)		CEV_P4a_3F	CAA CTG ACA ATG TAT CTC CAC C	Amplicon production for Sanger sequencing of batch 2022-A2 shipping water extract	Original
Conventional PCR		oPVP857	GTA CTT TAT TTG CTG CAG GAT	Amplicon production for Sanger sequencing of batch 2019-F1 shipping water extract and remaining 2020 and 2022 samples	[49]
		oPVP824	GTG GTA ACT TTA CTT GTC CTC C		

#### Phylogenetic analyses

Phylogenetic analyses were conducted using MEGA11 [35]. Only published partial *P4a* gene sequences that completely covered our shortest sequence (412 bp) were included for tree construction. All the sequences were therefore trimmed to 412 bp to match this sequence. A total of 110 sequences were analysed using the maximum likelihood method and the Tamura 3-parameter model (1000 bootstraps), with a gamma distribution of rates among sites and invariant sites.

### Statistical analysis

Statistical analysis was conducted using RStudio software and, unless otherwise specified, the embedded “stats” package [36, 37]. The Wilcoxon signed rank test, with continuity correction and paired data, was used to compare the CEV load between sample materials. Total extracted DNA concentrations were analysed for correlation with CEV genome copies using the Pearson correlation test. McNemar’s exact test (“exact2 × 2” package [38]) was utilised to compare positivity rates between different sample materials: shipping water versus fish bag swabs. For fish samples, logistic regression was performed to assess the association between CEV qPCR positivity (a binomial variable: CEV DNA detected or not) and carp qPCR Ct values (a continuous variable, since carp DNA was detected in all tested samples). The logistic model was fitted using the “glm” function with a binomial family.

### Evaluation of sensitivity and batch prevalence using latent class modelling

Latent class modelling was used to estimate the sensitivity of the different sampling methods at the batch level and the batch prevalence in the various imports. This approach is particularly useful when the true status of epidemiological units, the batches in our case, is unknown. Here, batch positivity was assessed through qPCR on different sample materials. Five sampling materials were tested: shipping water (200 µL + pellet), shipping water (1500 µL), fish bag swabs, pooled gill swabs, and gills of naturally deceased fish. For each batch, between one and four different sampling materials were used. Batches originated from three consignments, and a different batch prevalence was defined for each consignment.

The modelling was carried out using the R package “cmdstanr” [39] and was based on the “CIndep model” proposed by Keddie et al., which is a conditional independent model where every test is considered imperfect [40]*.* We modified this model to fit our case where certain tests were omitted for some batches, leading to eight different populations, each being tested with its own distinct combination of tests (cf. Additional file 2). The qPCR assays are assumed to have near perfect specificity: the prior distributions of the specificities were parameterised to follow a Beta (30,1) distribution, which means that 95% of these prior values were above 0.9. Other prior distributions (for sensitivities and prevalence) were considered uniform [40]. When multiple gill samples from the same batch were tested, the batch was considered positive if at least one sample tested positive (e.g., in batch 19-F1, the gill test was positive, as one of the three deceased fish tested positive).

All the models used are available in Additional file 2. We ran 4 simulation chains with 10 000 heating iterations and 2000 sampling iterations. Graphical results were generated using the “mcmc_areas” function from the “bayesplot” [41] package, “ggplot2” [42] and “ggpattern” [43].

## Results

### Detection of CEV in shipping water and fish bag swabs (2019, 2020 and 2022 shipments)

**Figure 1:**
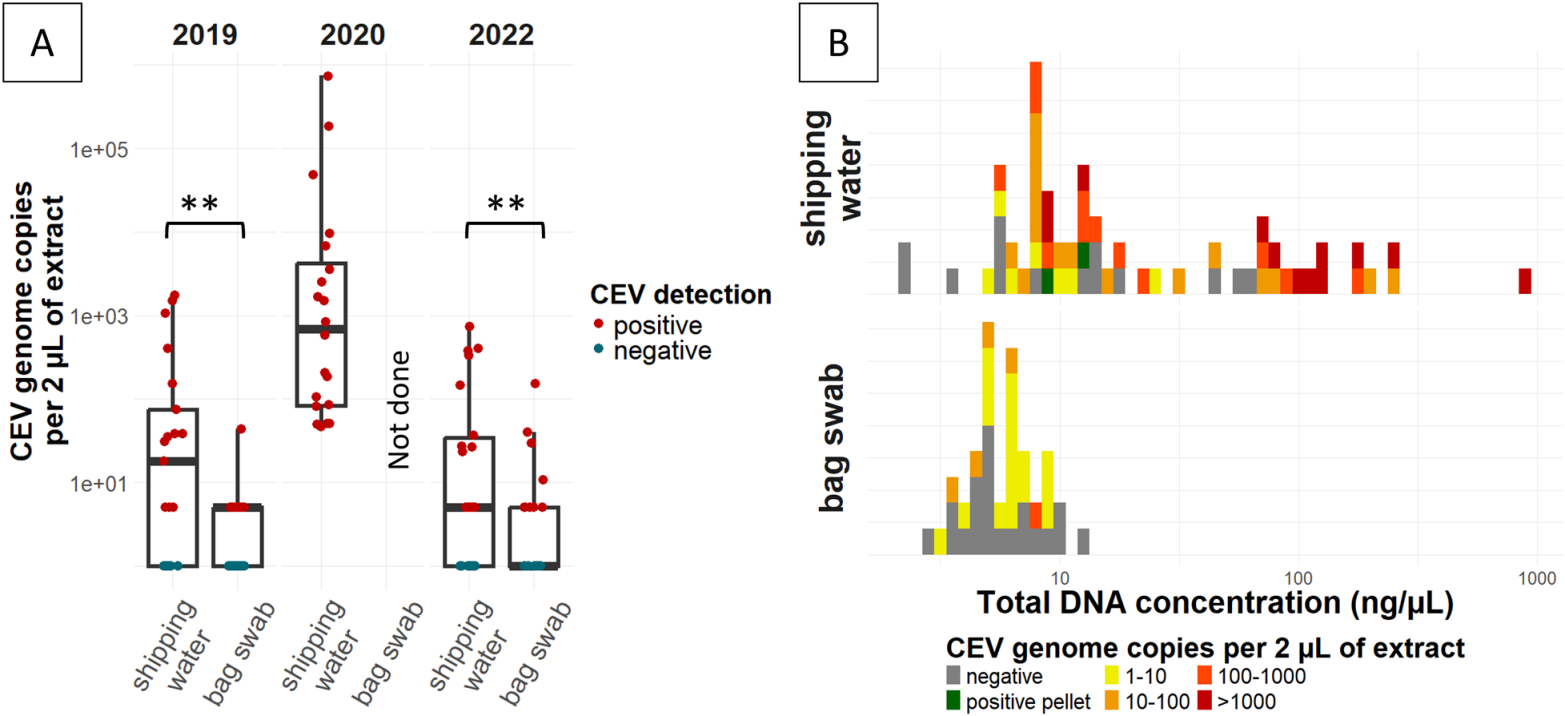
**CEV DNA load in shipping water and fish bag swabs. A** Comparison between shipping water and fish bag swabs collected in 2019, 2020 and 2022. Negative samples were displayed at 1 copy/2 µL for visual purposes on a logarithmic scale. **p < 0.01. **B** Distribution of the CEV load by total DNA concentration for shipping water across 2019, 2020 and 2022 shipments and for fish bag swabs in 2019 and 2022 shipments.

CEV DNA was detected in the majority of the shipping water samples. Specifically, for the 2019 shipment, 81% (17/21) of the samples contained the viral genome, whereas the frequency of detection reached 100% (20/20) for the 2020 shipment and 64% (14/22) for the 2022 shipment. CEV positivity was comparable between the fish bag swabs and the shipping water samples, with only two batches (19-D1 and 19-D3) testing positive in the fish bag swabs but not in the shipping water samples (Table 2). However, as shown in Figure 1A, the CEV genomic load was greater in the shipping water samples.

**Table 2:** **CEV-positivity of shipping environment samples**

Year of shipment	Shipping water positivity (including water pellet for 2019)	Fish bag swab positivity	Total positivity	Positivity comparison between shipping water and fish bag swab
2019	15/21 (71%)	12/21 (57%)	17/21 (81%)	Not significant (*p* = 0.45)
2020	20/20 (100%)	Not done	20/20 (100%)	Not applicable
2022	14/22 (64%)	10/22 (45%)	14/22 (64%)	Not significant (*p* = 0.13)

Because total DNA concentrations are often low, we examined whether negative samples were associated with the lowest DNA concentrations (Figure 1B). No correlation between the CEV load and the total DNA concentration was found for either the shipping water or the fish bag swabs, as indicated by the Pearson correlation test (*p* > 0.4).

### Detection of CEV in gill swabs and early deceased fish gills

#### CEV-negative shipping bag batches

**Figure 2:**
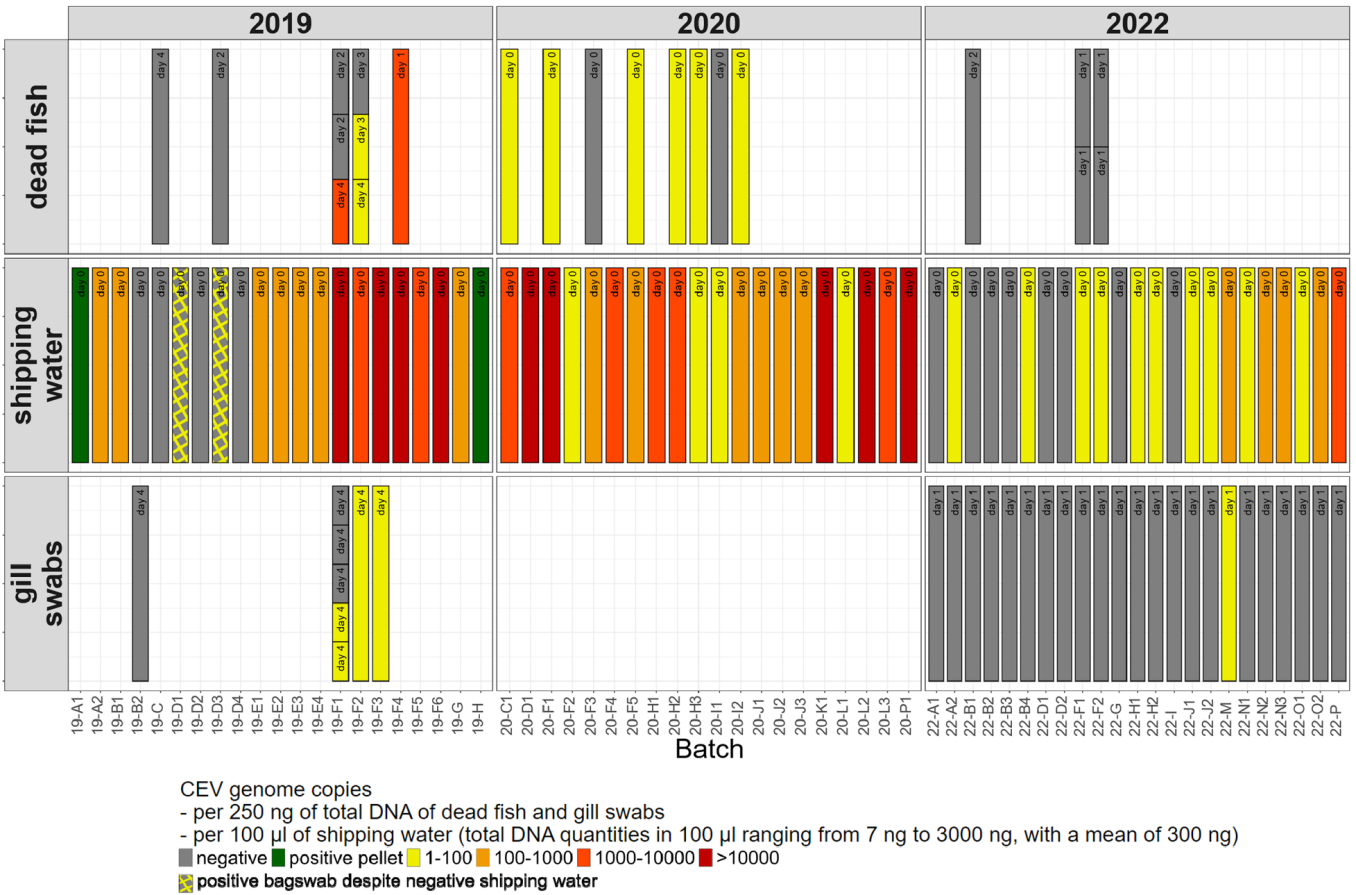
**CEV DNA detection in shipping bags and koi fish.** Sampling was conducted during three koi fish imports from Japan in 2019, 2020, and 2022. Dead fish were examined: gills of fish that were found dead in shipping bags on day 0 were analysed by a batch pool, whereas those that died in their aquaria between 1 and 4 days after arrival were individually analysed. Gill swabs were collected from five anaesthetised fish per batch and analysed in pools (except for batches 19-F1, which were analysed individually) four days after arrival in 2019 and within 12 h in 2022. The results of the fish bag swabs are not shown except when they were positive despite being negative for shipping water. Real-time PCR targeting carp DNA was used to confirm the amplification of DNA extracted from the fish samples. All tested samples, both CEV-negative and the majority of CEV-positive, were positive for carp DNA. Logistic regression analysis revealed no statistically significant association between CEV qPCR positivity and the Ct values of carp qPCR (*p* > 0.05).

CEV DNA was not detected in 4 out of 21 transport bags from the 2019 shipment (i.e., neither in shipping water, including water pellets, nor in fish bag swabs) nor in 8 out of 22 transport bags from the 2022 shipment. Gill swabs pooled from five samples per batch were performed on all CEV-negative batches in 2022 (22-A1, 22-B1, 22-B2, 22-B3, 22-D1, 22-D2, 22-G, and 22-J) and one batch in 2019 (19-B2); all tested negative for CEV (Figure 2). Notably, an individual from another CEV-negative batch (19-C) from the 2019 ship was found dead with no detectable CEV DNA in its gills 4 days after arrival, suggesting a cause of death unrelated to KSD.

#### CEV-positive shipping bag batches

CEV DNA was detected in most shipping bags: 17 out of 21 (81%) in 2019, 20 out of 20 (100%) in 2020 and 14 out of 22 (64%) in 2022 (Table 2).

For the 2020 shipment, several fish died during shipment across eight out of the 20 batches. Gills were pooled by batch for analysis, and CEV DNA was detected in the gills of six out of these eight batches and in the shipping water of all 20 batches (Figure 2).

For the 2019 and 2022 shipments, no fish died during shipment. However, within four days of arrival, eight fish from the 2019 shipment and four fish from the 2022 shipment, sourced from batches with CEV-positive water (from batches 19-D3, 19-F1, 19-F2, 19-F4, 22-F1 and 22-F2), were found dead. CEV DNA was detected in the gills of only four of the eight early deceased fish from the 2019 shipment (batches 19-F1, 19-F2 and 19-F4) and none of those from the 2022 shipment (Figure 2).

Gill swabs were collected four days after arrival from three batches (19-F1, 19-F2 and 19-F3) of shipping bags from the 2019 shipment that had tested positive for CEV. The five swabs per batch were analysed individually for batches 19-F1 and pooled for the other batches. Viral DNA was detected in all three batches, albeit at a low concentration (less than 10 copies/2 µL of extracted DNA). Two of the five gill swabs from batches 19-F1, which were individually analysed, tested positive for CEV. For 2022 shipment, gill swabs were collected from all batches, all of which tested positive for CEV in the shipping water. However, only one pool showed a positive result for CEV at a very low concentration (batch 22-M). This positive result was confirmed through electrophoresis of the qPCR product on an agarose gel, which revealed an expected product size of 200 bp (Figure 2).

### Influence of the shipping mortality rate on the shipping water and dead fish CEV loads

No fish died during transport in the 2019 and 2022 shipments. However, in the 2020 shipment, mortality rates during shipping ranged from 0 to 47%. CEV DNA was detected in the shipping water of every batch imported in 2020, with loads ranging from 8.10^1^ to 1.10^6^ copies per 100 µL of shipping water (total DNA quantities in 100 µL of shipping water ranged between 7 and 2.10^3^ ng, with a mean of 3.10^2^ ng). In contrast, the CEV load in the gills of fish that died during transport was either undetectable or low (< 10^2^ CEV genome copies per 250 ng of total DNA) (Figure 2).

**Figure 3:**
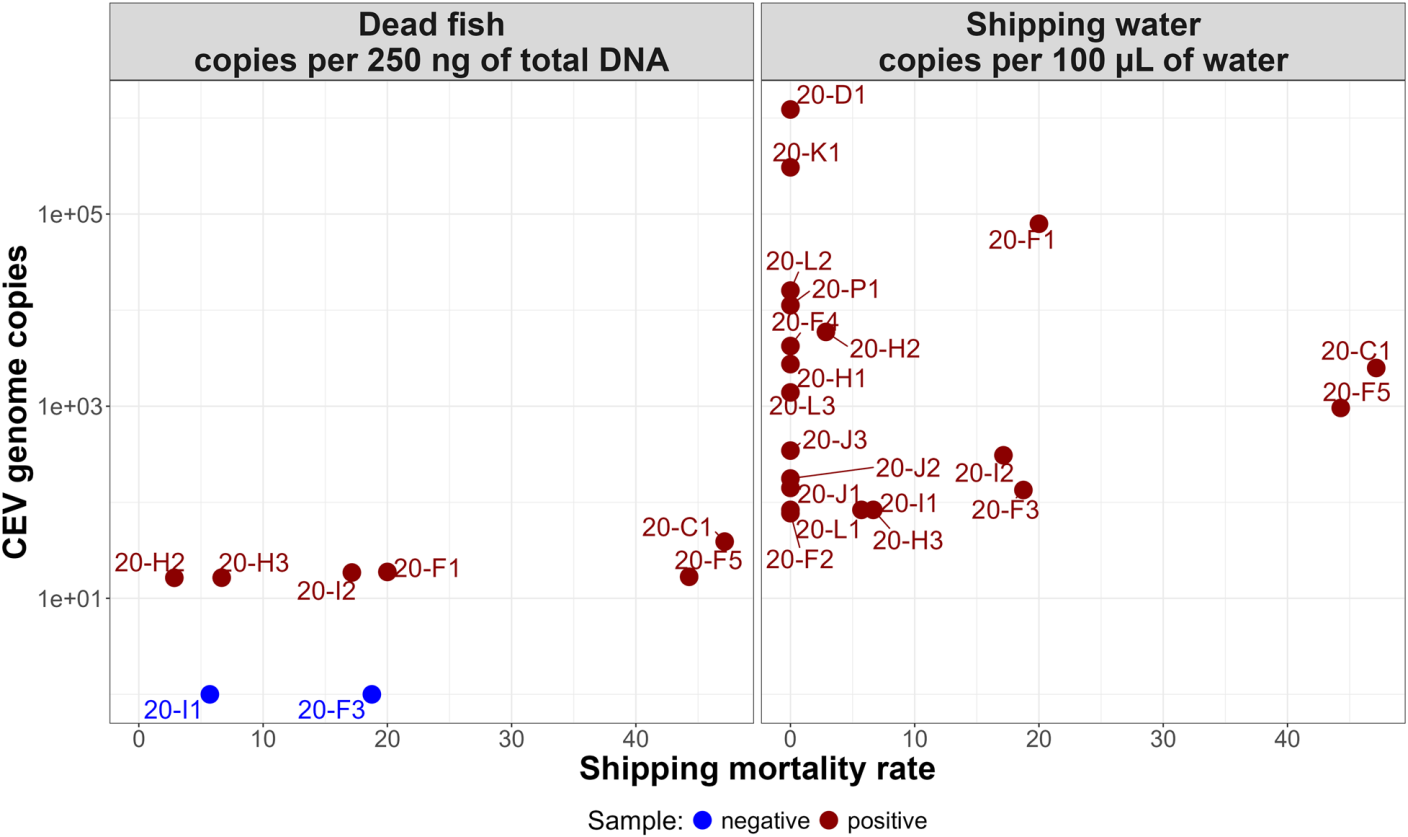
**Distribution of the CEV load in shipping bags and imported dead fish (pools by batch) according to the shipping mortality rate (restricted to the 2020 shipment).** The total DNA quantity in 100 µL of shipping water ranged between 7 and 2.10^3^ ng, with a mean of 3.10^2^ ng.

No correlation was detected between the CEV load and mortality rate, whether for gill pools or shipping water. Batches without shipping mortality presented both the lowest (20-L1) and the highest (20-D1) CEV loads in shipping water (Figure 3).

### Evaluation of sensitivity and batch prevalence using latent class modelling

**Figure 4:**
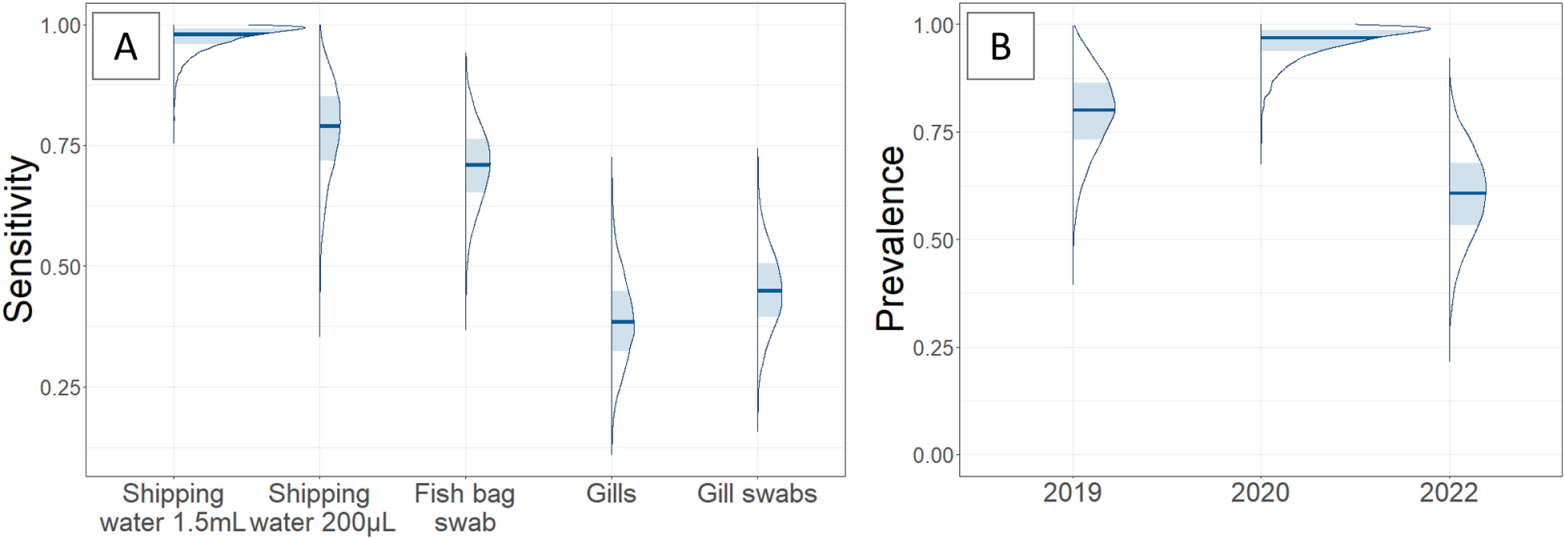
**Comparison of the estimated sensitivity of five sampling techniques used to detect CEV DNA from 64 Koi fish batches (A) and estimation of the prevalence among different imports (B) using latent class modelling.** The mass probability is represented by areas, with the shaded area representing 50% mass probability and the dark blue line representing the median. The various methods for sampling DNA include extracting it from 1.5 mL of shipping water and from 200 µL of shipping water (or negative from a 20 mL pellet), from a fish bag swab taken upon arrival, from the gills of naturally dead fish from 0 to 4 days after arrival, and from a pool of 5 gill swabs taken under anaesthesia from apparently healthy koi fish within 4 days of their arrival. The prevalence of DNA was estimated for each shipment studied, identified by their associated year.

As depicted in Figure 4A, the estimated sensitivity for CEV DNA detection in the context of koi fish import varies across the different sampling methods. The highest sensitivity was observed for sampling shipping water, with mean estimates of 98% (95% credible interval (CrI): 89–100%) when 1.5 mL of shipping water was sampled (2020 and 2022 shipments) and 78% (CrI: 57–94%) when 200 µL (± shipping water pellet) was sampled (2019 shipment). The sensitivity estimated for the fish bag swabs remained greater than that of the fish samples, with a mean estimate of 70% (CrI: 54–86%) for the bag swabs, 45% (CrI: 30–61%) for the pools of 5 gill swabs from non-symptomatic anaesthetised fish, and 39% (CrI: 22–57%) for the gills from fish that died naturally within 4 days of arrival.

The estimated batch prevalence of CEV DNA in imported koi fish (Figure 4B) varied between shipments, with a maximum mean of 96% (CrI: 84–100%) for the 2020 shipment and a minimum mean of 60% (CrI: 39–80%) for the 2022 shipment.

### Genetic diversity of imported CEV

Phylogenetic analyses were conducted to evaluate the diversity of viral variants introduced into France with Japanese koi and to determine whether the variants detected at the wholesaler facility shortly after and long after importation were the same as those imported. The shipping water samples were investigated primarily because they reflect the imported variants, having been collected before the release of the fish and, therefore, before any potential contamination from the receiving facility. From the 2019 shipment, nine CEV-positive samples originating from five different breeders were selected. A CEV-positive gill sample was also included from a koi that died within 24 h after arrival, making it unlikely that post-arrival contamination caused such rapid death. To compare haplotypes from the same breeder over time, we analysed four samples of shipping water from the 2020 and 2022 shipments. Additionally, we included eleven CEV-positive fish samples (dead fish gill or gill swabs) collected at least ten days after arrival from imported or resident batches to evaluate the diversity and origin of variants circulating in the French wholesaler facility beyond the immediate post-arrival period.

From these samples, we obtained 29 sequences that shared between 97.8 and 100% DNA identity, with a maximum of nine substitutions across 412 nt. Overall, all our sequences clustered within CEV genogroup II, as expected, since they were obtained from koi (Additional file 5). Our sequences presented between 0 and 14 substitutions compared with the published gII sequences available in GenBank, with a DNA identity of 96.6–100%. Compared with published gI sequences, our sequences presented between 17 and 29 substitutions, with a DNA identity ranging from 93.0 to 95.9%.

#### Variety of imported CEV sequences: shipping bags (day 0) and very early deceased fish (day 1)

**Figure 5:**
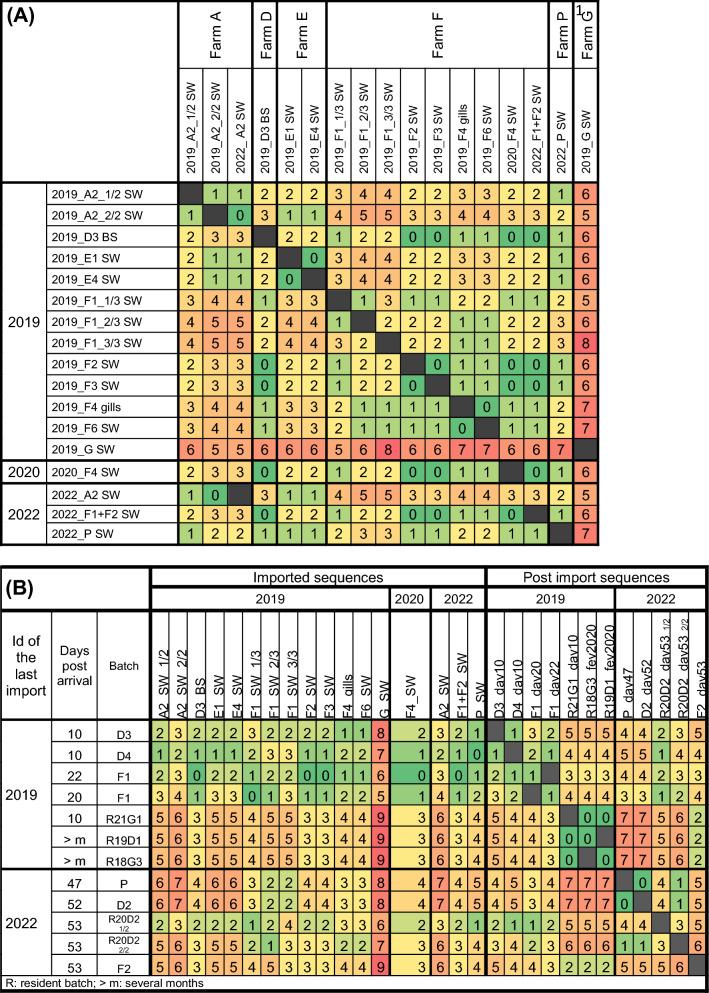
**Pairwise comparison (number of substitutions) of sequences (A) from the shipping environment and very early deceased fish and (B) from major post-import outbreaks.** SW: shipping water; BS: fishbag swab; gills: gills of naturally dead fish. The greener the color of the cell is, the fewer substitutions are present; the redder the color is, the more substitutions are observed.

A total of 17 sequences were generated from shipping water samples, fish bag swabs, and very early deceased fish from the three imports studied. For some farmers (i.e., farmers A, E, P, and G), no identical sequence was found in samples from other farmers, suggesting an association between the farmer and the detected sequences (Figure 5A). Additionally, some elements suggested possible genetic stability of variants circulating within these farms: (1) identical sequences were found in batches 19-E1 and 19-E4, as well as in 19-A2 and 22-A2 which were imported three years apart; (2) the number of detected variants in these batches was very low, with no variants detected in most, except for batches 19-A2, which presented two sequences differing by only one substitution (Figure 5A).

In contrast, relatively high intra- and inter-batch diversity was observed in farmer F's samples, with an identical sequence shared between shipping environment samples from batches originating from farmers F and D (i.e., 19-F2, 19-F3, and 19-D3) (Figure 5A). Although the maximum number of substitutions between farmer F sequences was only three, a total of five different sequences were detected across the 7 tested batches between 2019 and 2022. Notably, three different variants, differing by one to three substitutions, were detected in a single sample: shipping water from batches 19-F1.

#### Sequences detected beyond import (from day 10 to several months after import)

These sequences were detected either in non-clinical, recently imported fish (19-D3 and 19-D4) or in samples collected during KSD outbreaks. CEV sequences detected in 19-D3 and 19-D4 gill swabs ten days after import did not show 100% identity with any 2019 imported sequences (Figure 5B), making it impossible to draw conclusions about the origin of CEV circulation in these two batches. However, the two sequences from batches 19-F1, both detected in fish that died 20 and 22 days after arrival, were identical to those imported from farmer F (Figure 5B).

Notably, the sequence detected in the resident batch 21-G1, which exhibited clinical signs of KSD, differed by 4–5 substitutions from the sequences detected in batches 19-D3 and 19-D4, which were, however, collected the same day, as well as from all 2019 imported sequences (Figure 5B). In February 2020, a major CEV outbreak occurred in the whole facility, resulting in fish mortality. The same sequence was detected in two samples from different tanks (19-D1 and 18-G3). Interestingly, this sequence was 100% identical to the one detected in the resident batch 21-G1 mentioned above, i.e., 10 months earlier (Figure 5A).

Approximately 6 weeks after the 2022 import, another major CEV outbreak occurred. Four samples were sequenced between 47 and 53 days after import from both imported and resident batches (Figure 5B). While the same sequence was detected in two samples from different batches (22-D and 22-P), this outbreak was characterised by multiple circulating variants (at least four). Indeed, two variants were detected in the gills of a single dead fish (resident batch 20-D2), and another sequence was detected in the gills of a fish from batch 22-F2 (Figure 5B).

## Discussion

Systematic screening is essential for implementing effective control measures against the spread of pathogens, including CEV. For imported koi carp, this screening method must be simple, cost effective, and nonlethal. To reduce costs, one approach is to minimise the number of tests by using representative environmental samples or pooled individual tests. However, this approach may decrease sensitivity.

This study compared the results of qPCR performed on various sample materials, including shipping water, fish bag swabs, pools of gill swabs from anaesthetised fish, and gills from naturally deceased fish. The latent class analysis revealed that the environment samples had higher sensitivity than the fish samples. Specifically, the highest sensitivity was associated with 1.5 mL of shipping water, with a 95% credible interval above 89%. In contrast, the 95% confidence intervals of the sensitivities associated with gills from naturally deceased fish and pools of five gill swabs from anaesthetised fish were both less than 61%. These results emphasised the importance of sampling before eliminating the water and releasing the fish into the farm. Moreover, collecting shipping water or bag swabs is easy, non-invasive for fish and cost-effective, as only one test per batch is needed.

The presence of many fish samples (gill swabs and gills of naturally deceased fish) with low positive results (< 10^2^ CEV genome copies/250 ng of total DNA) or negative results, while the shipping water tested positive for CEV DNA, was unexpected. There are three possible explanations for the weak detection of CEV DNA in koi fish gills and gill swabs: (1) the possibility of sampling bias, particularly if only a small number of fish are infected or if the fish are no longer shedding the virus at the time of sampling; (2) a possible shift in viral tropism from gills to other organs in asymptomatic shedders compared with experimentally infected fish; and (3) the possibility that death is not due to CEV but rather to other stressors such as shipping density, changes in water chemistry, temperature changes, potential oxygen depletion or other concurrent diseases [44], which strengthen the sampling bias. Interestingly, a recent study revealed that when common carp fry (45–50 g) were infected through cohabitation with CEV-infected fish, no mortality occurred, and a shift in tropism was observed, with viral loads being greater in the skin than in the gills, although overall viral loads were low [45]. Despite key differences between the experimentally infected common carp fry and the young imported koi fish, such as the host, viral strain, water temperature and probable long-term exposure of koi to CEV, certain similarities, such as the absence of mortality and young age of the carp, could suggest a similar tropism shift in imported koi, explaining why most of the gills were negative but water positive, possibly due to viral shedding in the cutaneous mucus. Unfortunately, we were unable to test this hypothesis, as only the gills were collected and stored. Furthermore, when there are viral shedders in a batch, it is theoretically possible to detect viral DNA. The probability of detection is then correlated with the number of shedders, not the size of the population [46]. In the koi industry, even if the within-batch prevalence is low, the probability of having at least one shedder is relatively high, as batches typically consist of fifty to hundreds of individuals. This can explain the high predicted sensitivity associated with 1.5 mL shipping water sampling.

Another advantage of shipping water is that it can be collected before the release of the fish into their tanks or ponds. If processed quickly, it would be possible to adapt each batch’s quarantine procedure on the basis of its CEV status. Unfortunately, to our knowledge, such a rapid detection test for CEV is not yet commercially available. However, testing shipping water allowed us to confirm that the detected DNA was imported and not due to post-importation contamination of the fish. When combined with phylogenetic analyses, this approach may help in understanding how CEV circulates at both the international and wholesaler levels.

In the present study, CEV DNA was detected in 81% (51/63) of the imported batches of the shipping environment samples. The estimated batch prevalence through latent class modelling was high. For 2019 imports, the mass probability mean was 79%; for 2020 imports, it was 95%; and for 2022 imports, it was 60%. All of these estimates had 95% confidence intervals above 40%. This high proportion of CEV DNA detection was unexpected in freshly imported koi carp, since most batches appeared healthy with low or no mortality. These results highlight the importance of conducting systematic surveillance on all batches, rather than focusing solely on clinical cases, to monitor CEV effectively.

However, the detection of CEV DNA does not necessarily mean that infectious particles were packed on the farm and shipped to France; it could be free of environmental CEV DNA or DNA embedded in non-infectious particles. This mismatch is known, although poorly documented in the fish trade. For example, the detection of the fish parasite *Neobenedenia girellae* DNA in fish shipping bags after an experimental infection was explored under different scenarios. The study revealed two main outcomes: (1) limited sensitivity, with only 50% of infected fish transported in clean water testing positive, and (2) a lack of specificity, with 23% of treated fish (with dead parasites) in clean water and 70% of noninfected fish in contaminated water testing positive [47]. Similarly, in the case of the CEV, non-shedding carriers could be packed in clean water and not resume shedding during transport, leading to lower-than-expected sensitivity. Conversely, the presence of CEV DNA in the water indicates that the batch originated from facilities where the virus was present. Given the high contagiousness of CEV, it is very likely that at least some fish were infected [30]. We assume that the specificity for detecting CEV DNA is much greater than that for detecting parasites, reflecting the true status of the batch. Furthermore, several findings suggest that viable CEV was imported with some koi fish: (1) high viral DNA loads (up to 1.2 × 10^6^ CEV genome copies/100 µL of shipping water) and high detection frequency in shipping water; (2) the death of a CEV-infected koi fish, with a high CEV DNA load (batch 19-F5, 4.7 × 10^3^ CEV genome copies per 250 ng total DNA) less than 24 h after its arrival, which is inconsistent with a post-arrival infection; and (3) the detection of the same *P4a* variant in both shipping water and a koi fish that died 20 days after its arrival (batch 19-F1). Nevertheless, to accurately assess the sensitivity and specificity of qPCR assays, experimental infections would be helpful.

The use of shipping water allowed us to genetically characterise the variants that were imported without any possibility of post-arrival fish infection. As expected, all sequences clustered in genogroup II. Interestingly, it seems that some breeders are associated with specific sequences (e.g*.,* Farmer E). These sequences were also detected in Japan and other French facilities in 2019, suggesting repeated dissemination from a common centre. However, this observation is based on very few SNP carried out on a limited portion of the genome. On the other hand, we observed some identical sequences in batches from different breeders (e.g*.*, Farms D, F and P) and that many different sequences can be found in batches from only one farmer and even in a unique batch (e.g*.,* Farm F). We assume that this could be linked to farmers’ connections through koi exchanges, common stocking before export, or hydrologic connections. Assessing this hypothesis requires performing phylogenetic analyses on much larger sequences. Further research is needed to confirm or invalidate the putative links between particular variants and farmers.

To reduce the incidence of clinical signs of KSD, the koi batches in the present study were subjected to salt bathing upon arrival and for several days. This treatment has been used by farmers for decades for this purpose [7, 26, 48]. KSD outbreaks have occurred in recently imported (after the end of salt treatment) and resident batches, without any introduction into the greenhouse, from weeks to several months after koi import. These findings suggest that some koi fish may carry the CEV asymptomatically for weeks or months.

Sanger sequencing carried out for these outbreaks did not reveal a clear dissemination pattern within the wholesaler facilities. In February 2020, it appeared that only one variant was disseminated. This was evidenced by the fact that two sequences from two different resident batches were 100% identical to one sequence from a fish in another resident batch that had died 10 days after the April 2019 shipping (i.e., months earlier). It is believed that an asymptomatic carrier resumed shedding of the CEV, thereby infecting other fish in its own tank and subsequently spreading the virus throughout the greenhouse.

Approximately 2 months after the 2022 importation, several variants circulated among both resident and imported batches. The mixes of variants varied between batches, indicating that multiple asymptomatic carriers may have started shedding the virus again simultaneously, potentially due to a stressful event. As previously stated, a stressful event can trigger a KSD outbreak [3]. The high batch prevalence of shipping water samples that tested positive for CEV might also be due to asymptomatic carriers who resumed shedding during the stressful event of packing and shipping.

Interestingly, some fish originating from batches in which CEV DNA was detected in shipping water (e.g*.,* 22-F2) died of CEV infection during this outbreak. This finding shows that a batch with CEV-positive shipping water cannot be considered protected against CEV infection.

The koi carp trade serves as a route for the spread of CEV, with a high prevalence of positive imported batches. The presence of the same variants in transport bags and in mortality outbreaks indicates that the infectious virus has been imported, at least in certain batches. Additionally, even if the batches appear healthy, they can cause mortality outbreaks several months after arrival.

With respect to detection methods, environmental samples from shipping, such as shipping water and fish bag swabs, are suitable for CEV surveillance in the koi trade. This approach reliably identifies virus-positive batches without the need to test the fish directly. It also demonstrated a high predicted sensitivity (95% confidence interval above 89%) when DNA was extracted from 1.5 mL. While the estimated sensitivity for fish bag swabs is lower, it remains higher than that for fish sampling, which has a predicted sensitivity with a 95% credible interval below 61%. Water collection is easy for farmers and non-invasive for fish. Furthermore, it is feasible to collect transport water before new fish are introduced into facilities to enhance biosecurity measures.

## Supplementary Information

**Additional file 1. Protocols for CEV detection by qPCR and** ***P4a*** **partial cds Sanger sequencing.**

**Additional file 2. Format data and R script for latent class modelling**.

**Additional file 3. GenBank references of the sequences produced in the present work**.

**Additional file 4. qPCR results produced in the present work**.

**Additional file 5. Phylogenetic analyses of a 412-nt fragment of the** ***P4a*** **gene in imported, resident koi fish batches and published sequences** (***n* = 110). **

## Data Availability

The sequence datasets generated during this study are available in the GenBank repository at https://www.ncbi.nlm.nih.gov/genbank/ (see Additional file 3). The datasets used and/or analysed in this study are available from the corresponding author upon reasonable request.
