# Supplementary material for: Carp edema virus surveillance in the koi trade: early detection through shipping environment sampling and longitudinal monitoring of CEV outbreaks in a wholesaler facility
Source: Vet Res. 2025 Mar 4;56:48. doi: 10.1186/s13567-025-01476-1 (PMC11881292; doi:10.1186/s13567-025-01476-1)
Supplement: Supplementary file 1 — Additional file 1. Protocols for CEV detection by qPCR and P4a partial cds Sanger sequencing. [file 13567_2025_1476_MOESM1_ESM.docx]

# **Additional file 1: Protocols for CEV detection by qPCR and *P4a* Partial cds Sanger sequencing**

Primer sequences are provided in the main body of the article.

- qPCR targeting CEV *P4a* gene

The qPCR reaction mixture included 1X iTaqTM Universal SYBR®Green Supermix (Bio Rad Laboratories Inc.), 200 nM of each primer (CEV_TiHo_qF and CEV_TiHo_qR), 2 µL of template, and water up to 10 µL. All qPCRs were performed in duplicates for each DNA sample. Thermal cycling conditions consisted of an initial denaturation at 95 °C for 120-200 s, followed by 45 cycles at 95 °C for 15 s, 58 °C for 30 s, and a final melting phase (95 °C for 5 s, 65 °C for 60 s, and 97 °C for 1 s). The SYBR®Green fluorescence intensity was measured during each elongation cycle and melting phase.

- DNA amplification for Sanger sequencing (PCR +/- cloning)
  - **Protocol for 2019 & February 2020 samples.**

A 528 or 478 bp fragment of the *P4a* gene was targeted using the nested PCR method as developed by CEFAS for amplicon production. The PCR reaction included of 1× Kapa taq buffer (Sigma Aldrich) in a 50 μL reaction volume, along with 2 µL of 25 mM MgCl2, 1 µL of 10 mM dNTP mix, 2.5 µL of 10 µM primers CEV_for_B and CEV_rev_J, 1.0 µL of Kapa taq, 28 µL of water, and 4 µL of DNA extract (diluted as for qPCR). The reaction underwent 40 cycles: 30 s at 95 °C, 1 min at 55 °C, and 30 s at 72 °C following an initial denaturing step of 2 minutes at 95 °C. A final extension step of 30 s at 72 °C concluded the process. Subsequently, 5 µL of first-round amplification products were electrophoresed in a 1% agarose gel with 1X SYBR® Safe gel DNA stain (Invitrogen) in 0.5 X TBE (Tris-Borate-EDTA) buffer. A second round of nested PCR was conducted only if a band indicating PCR product was either absent or weak. The PCR used the same conditions as the first round, and was performed using primers CEV_For_B_int and CEV_Rev_J_int. The first and second round products’ remaining samples were subsequently separated in a 2% agarose gel whereby the product band was excised and purified with the Nucleospin® gel and PCR clean up kit (Macherey Nagel, Germany). The purified amplicons were sequenced using SANGER technology in both directions.

- - **Protocol for shipping water of batch 22-A2.**

A semi-nested PCR was carried out on this sample, targeting a larger *P4a* gene fragment. The reaction mixture included 1x Coral Load Buffer (Qiagen, Netherlands), 0.4 µL of 10 nM dNTP, 1.0 µL of each 10 µM primer (1st round: primers CEV_P4a_3F and CEV_rev_J; 2nd round: primers CEV_P4a_3F and CEV_rev_J_int), 0.2 µL of Taq polymerase, 11.4 µL of water, and 4.0 µL of DNA extract or first-round product. Each round consisted of an initial denaturation step (2 min at 95 °C), 35 cycles: 30 s at 95 °C, 30 s at 50 °C, and 1 min at 72 °C, and a final extension step of 1 min at 72 °C. The PCR product was then separated on a 1% agarose gel, cleaned up and Sanger sequenced as previously described.

- - **Protocol for shipping water of batch 19-F1 and remaining 2020 and 2022 samples**

A region of the *P4a* ORF was amplified using a newly designed set of primers (oPVP824/oPVP857). The amplified portion varied between 1074 and 1077bp and encompasses the portion of 890-893 bp previously published by the ANSES Laboratory. Each primer was used at 400 nM in the final PCR reaction, which included SuperScriptTM Platinum High Fidelity (Invitrogen) and co-reactants, as previously described by Baud et al. [3]. The PCR cycles consisted of one denaturation step at 94 °C for 2 min, followed by 40 cycles at 94 °C for 15 s, 58 °C for 30 s and 68 °C for 1 min. Amplicons were TA-cloned using a kit (TOPO-TA, Invitrogen).

Sequencing was carried out at the ANSES Laboratory, as reported previously [3], or by the commercial GATC sequencing service (Eurofins). In some cases, several amplicon clones from a single sample were sequenced.
