# Supplementary material for: Carp edema virus surveillance in the koi trade: early detection through shipping environment sampling and longitudinal monitoring of CEV outbreaks in a wholesaler facility
Source: Vet Res. 2025 Mar 4;56:48. doi: 10.1186/s13567-025-01476-1 (PMC11881292; doi:10.1186/s13567-025-01476-1)
Supplement: Supplementary file 2 — Additional file 2. Format data and R script for latent class modelling. [file 13567_2025_1476_MOESM2_ESM.docx]

# **Additional file 2: Format data and R script for latent class modelling**

Data:

Data is compiled into a list labelled “data”, containing the results of all tests (‘pxty’) as specified in the accompanying table. The list also includes the size of each population (Nx) and the number of distinct tests performed on each population (Mx).

|  | Test 0: Shipping water (200 µL) | Test 1: Shipping water (1.5 mL) | Test 2: Fish bag swab | Test 3: Gills | Test 4:  Gill swabs | Year of the import |
| --- | --- | --- | --- | --- | --- | --- |
| Population 1 |  | p1t1 | p1t2 | p1t3 | p1t4 | 2022 |
| Population 2 |  | p2t1 | p2t2 |  | p2t4 | 2022 |
| Population 3 | p3t0 |  | p3t2 | p3t3 |  | 2019 |
| Population 4 | p4t0 |  | p4t0 |  |  | 2019 |
| Population 5 |  | p5t1 |  | p5t3 |  | 2020 |
| Population 6 | p6t0 |  | p6t2 | p6t3 | p6t4 | 2019 |
| Population 7 | p7t0 |  | p7t2 |  | p7t4 | 2019 |
| Population 8 |  | p8t1 |  |  |  | 2020 |

Script:

library(cmdstanr)

#program description and compilation

stan_program_8_pop <- write_stan_file(

"data {

int<lower=1> M1; // Number of different tests in population 1

int<lower=1> N1; // Size of population 1

array[N1] int<lower=0, upper=1> p1t1; // results of test number 1

array[N1] int<lower=0, upper=1> p1t2;

array[N1] int<lower=0, upper=1> p1t3;

array[N1] int<lower=0, upper=1> p1t4;

int<lower=1> M2;

int<lower=1> N2;

array[N2] int<lower=0, upper=1> p2t1;

array[N2] int<lower=0, upper=1> p2t2;

array[N2] int<lower=0, upper=1> p2t4;

int<lower=1> M3;

int<lower=1> N3;

array[N3] int<lower=0, upper=1> p3t0;

array[N3] int<lower=0, upper=1> p3t2;

array[N3] int<lower=0, upper=1> p3t3;

int<lower=1> M4;

int<lower=1> N4;

array[N4] int<lower=0, upper=1> p4t0;

array[N4] int<lower=0, upper=1> p4t2;

int<lower=1> M5;

int<lower=1> N5;

array[N5] int<lower=0, upper=1> p5t1;

array[N5] int<lower=0, upper=1> p5t3;

int<lower=1> M6;

int<lower=1> N6;

array[N6] int<lower=0, upper=1> p6t0;

array[N6] int<lower=0, upper=1> p6t2;

array[N6] int<lower=0, upper=1> p6t3;

array[N6] int<lower=0, upper=1> p6t4;

int<lower=1> M7;

int<lower=1> N7;

array[N7] int<lower=0, upper=1> p7t0;

array[N7] int<lower=0, upper=1> p7t2;

array[N7] int<lower=0, upper=1> p7t4;

int<lower=1> M8;

int<lower=1> N8;

array[N8] int<lower=0, upper=1> p8t1;

}

parameters {

real<lower=0,upper=1> prev19; // prevalence import 2019

real<lower=0,upper=1> prev20; // prevalence import 2020

real<lower=0,upper=1> prev22; // prevalence import 2022

real<lower=0,upper=1> a01; // specificity test 0

real<lower=1-inv_logit(logit(a01)*2),upper=1> a02; // sensitivity test 0

real<lower=0,upper=1> a11; // specificity test 1

real<lower=1-inv_logit(logit(a11)*2),upper=1> a12; // sensitivity test 1

real<lower=0,upper=1> a21; // specificity test 2

real<lower=1-inv_logit(logit(a21)*2),upper=1> a22; // sensitivity test 2

real<lower=0,upper=1> a31; // specificity test 3

real<lower=1-inv_logit(logit(a31)*2),upper=1> a32; // sensitivity test 3

real<lower=0,upper=1> a41; // specificity test 4

real<lower=1-inv_logit(logit(a41)*2),upper=1> a42; // sensitivity test 4

}

transformed parameters {

simplex[2] theta19; // probability of being infected or not infected for year 2019

simplex[2] theta20; // probability of being infected or not infected for year 2020

simplex[2] theta22; // probability of being infected or not infected for year 2022

array[M1,2] vector[N1] prob1;

array[M2,2] vector[N2] prob2;

array[M3,2] vector[N3] prob3;

array[M4,2] vector[N4] prob4;

array[M5,2] vector[N5] prob5;

array[M6,2] vector[N6] prob6;

array[M7,2] vector[N7] prob7;

array[M8,2] vector[N8] prob8;

theta19[1] = 1-prev19;

theta19[2] = prev19;

theta20[1] = 1-prev20;

theta20[2] = prev20;

theta22[1] = 1-prev22;

theta22[2] = prev22;

prob1[1,1] = rep_vector(1-a11, N1);

prob1[1,2] = rep_vector(a12,N1);

prob1[2,1] = rep_vector(1-a21,N1);

prob1[2,2] = rep_vector(a22,N1);

prob1[3,1] = rep_vector(1-a31,N1);

prob1[3,2] = rep_vector(a32,N1);

prob1[4,1] = rep_vector(1-a41,N1);

prob1[4,2] = rep_vector(a42,N1);

prob2[1,1] = rep_vector(1-a11,N2);

prob2[1,2] = rep_vector(a12,N2);

prob2[2,1] = rep_vector(1-a21,N2);

prob2[2,2] = rep_vector(a22,N2);

prob2[3,1] = rep_vector(1-a31,N2);

prob2[3,2] = rep_vector(a32,N2);

prob3[1,1] = rep_vector(1-a01,N3);

prob3[1,2] = rep_vector(a02,N3);

prob3[2,1] = rep_vector(1-a21,N3);

prob3[2,2] = rep_vector(a22,N3);

prob3[3,1] = rep_vector(1-a31,N3);

prob3[3,2] = rep_vector(a32,N3);

prob4[1,1] = rep_vector(1-a01,N4);

prob4[1,2] = rep_vector(a02,N4);

prob4[2,1] = rep_vector(1-a21,N4);

prob4[2,2] = rep_vector(a22,N4);

prob5[1,1] = rep_vector(1-a11,N5);

prob5[1,2] = rep_vector(a12,N5);

prob5[2,1] = rep_vector(1-a31,N5);

prob5[2,2] = rep_vector(a32,N5);

prob6[1,1] = rep_vector(1-a01,N6);

prob6[1,2] = rep_vector(a02,N6);

prob6[2,1] = rep_vector(1-a21,N6);

prob6[2,2] = rep_vector(a22,N6);

prob6[3,1] = rep_vector(1-a31,N6);

prob6[3,2] = rep_vector(a32,N6);

prob6[4,1] = rep_vector(1-a41,N6);

prob6[4,2] = rep_vector(a42,N6);

prob7[1,1] = rep_vector(1-a01,N7);

prob7[1,2] = rep_vector(a02,N7);

prob7[2,1] = rep_vector(1-a21,N7);

prob7[2,2] = rep_vector(a22,N7);

prob7[3,1] = rep_vector(1-a41,N7);

prob7[3,2] = rep_vector(a42,N7);

prob8[1,1] = rep_vector(1-a11,N8);

prob8[1,2] = rep_vector(a12,N8);

}

model {

array[2] real ps;

// priors

a01~beta(30,1);

a02~beta(1,1);

a11~beta(30,1);

a12~beta(1,1);

a21~beta(30,1);

a22~beta(1,1);

a31~beta(30,1);

a32~beta(1,1);

a41~beta(30,1);

a42~beta(1,1);

prev19~beta(1,1);

prev20~beta(1,1);

prev22~beta(1,1);

//loop for population 1 (year 2022)

for(n in 1:N1){

for(k in 1:2){

ps[k] = log(theta22[k]) + binomial_lpmf(p1t1[n]| 1, prob1[1,k,n]) + binomial_lpmf(p1t2[n]| 1, prob1[2,k,n]) + binomial_lpmf(p1t3[n]| 1, prob1[3,k,n]) + binomial_lpmf(p1t4[n]| 1, prob1[4,k,n]);

}

target += log_sum_exp(ps);

}

//loop for population 2 (year 2022)

for(n in 1:N2){

for(k in 1:2){

ps[k] = log(theta22[k]) + binomial_lpmf(p2t1[n]| 1, prob2[1,k,n]) + binomial_lpmf(p2t2[n]| 1, prob2[2,k,n]) + binomial_lpmf(p2t4[n]| 1, prob2[3,k,n]);

}

target += log_sum_exp(ps);

}

// loop for population 3 (year 2019)

for(n in 1:N3){

for(k in 1:2){

ps[k] = log(theta19[k]) + binomial_lpmf(p3t0[n]| 1, prob3[1,k,n]) + binomial_lpmf(p3t2[n]| 1, prob3[2,k,n]) + binomial_lpmf(p3t3[n]| 1, prob3[3,k,n]);

}

target += log_sum_exp(ps);

}

// loop for population 4 (year 2019)

for(n in 1:N4){

for(k in 1:2){

ps[k] = log(theta19[k]) + binomial_lpmf(p4t0[n]| 1, prob4[1,k,n]) + binomial_lpmf(p4t2[n]| 1, prob4[2,k,n]);

}

target += log_sum_exp(ps);

}

//loop for population 5 (year 2020)

for(n in 1:N5){

for(k in 1:2){

ps[k] = log(theta20[k]) + binomial_lpmf(p5t1[n]| 1, prob5[1,k,n]) + binomial_lpmf(p5t3[n]| 1, prob5[2,k,n]);

}

target += log_sum_exp(ps);

}

//loop for population 6 (year 2019)

for(n in 1:N6){

for(k in 1:2){

ps[k] = log(theta19[k]) + binomial_lpmf(p6t0[n]| 1, prob6[1,k,n]) + binomial_lpmf(p6t2[n]| 1, prob6[2,k,n]) + binomial_lpmf(p6t3[n]| 1, prob6[3,k,n]) + binomial_lpmf(p6t4[n]| 1, prob6[4,k,n]);

}

target += log_sum_exp(ps);

}

//loop for population 7 (year 2019)

for(n in 1:N7){

for(k in 1:2){

ps[k] = log(theta19[k]) + binomial_lpmf(p7t0[n]| 1, prob7[1,k,n]) + binomial_lpmf(p7t2[n]| 1, prob7[2,k,n]) + binomial_lpmf(p7t4[n]| 1, prob7[3,k,n]);

}

target += log_sum_exp(ps);

}

//loop for population 8 (year 2020)

for(n in 1:N8){

for(k in 1:2){

ps[k] = log(theta20[k]) + binomial_lpmf(p8t1[n]| 1, prob8[1,k,n]);

}

target += log_sum_exp(ps);

}

}

generated quantities {

array[M1] real Se_mean1;

array[M1] real Sp_mean1;

array[M2] real Se_mean2;

array[M2] real Sp_mean2;

array[M3] real Se_mean3;

array[M3] real Sp_mean3;

array[M4] real Se_mean4;

array[M4] real Sp_mean4;

array[M5] real Se_mean5;

array[M5] real Sp_mean5;

array[M6] real Se_mean6;

array[M6] real Sp_mean6;

array[M7] real Se_mean7;

array[M7] real Sp_mean7;

array[M8] real Se_mean8;

array[M8] real Sp_mean8;

real Sensitivity_SW1500;

real Sensitivity_SW200;

real Sensitivity_BS;

real Sensitivity_gills;

real Sensitivity_GS;

real Specificity_SW1500;

real Specificity_SW200;

real Specificity_BS;

real Specificity_gills;

real Specificity_GS;

for(m in 1:M1){

Se_mean1[m] = mean(prob1[m,2,]);

Sp_mean1[m] = mean(1-prob1[m,1,]);

}

for(m in 1:M2){

Se_mean2[m] = mean(prob2[m,2,]);

Sp_mean2[m] = mean(1-prob2[m,1,]);

}

for(m in 1:M3){

Se_mean3[m] = mean(prob3[m,2,]);

Sp_mean3[m] = mean(1-prob3[m,1,]);

}

for(m in 1:M4){

Se_mean4[m] = mean(prob4[m,2,]);

Sp_mean4[m] = mean(1-prob4[m,1,]);

}

for(m in 1:M5){

Se_mean5[m] = mean(prob5[m,2,]);

Sp_mean5[m] = mean(1-prob5[m,1,]);

}

for(m in 1:M6){

Se_mean6[m] = mean(prob6[m,2,]);

Sp_mean6[m] = mean(1-prob6[m,1,]);

}

for(m in 1:M7){

Se_mean7[m] = mean(prob7[m,2,]);

Sp_mean7[m] = mean(1-prob7[m,1,]);

}

for(m in 1:M8){

Se_mean8[m] = mean(prob8[m,2,]);

Sp_mean8[m] = mean(1-prob8[m,1,]);

}

Sensitivity_SW1500 = (Se_mean1[1]*N1 + Se_mean2[1]*N2 + Se_mean5[1]*N5 + Se_mean8[1]*N8)/(N1 + N2 + N5 + N8);

Sensitivity_SW200 = (Se_mean3[1]*N3 + Se_mean4[1]*N4 + Se_mean6[1]*N6 + Se_mean7[1]*N7)/(N3 + N4 + N6 + N7);

Sensitivity_BS = (Se_mean1[2]*N1 + Se_mean2[2]*N2 + Se_mean3[2]*N3 + Se_mean4[2]*N4 + Se_mean6[2]*N6 + Se_mean7[2]*N7)/(N1 + N2 + N3 + N4 + N6 + N7);

Sensitivity_gills = (Se_mean1[3]*N1 + Se_mean3[3]*N3 + Se_mean5[2]*N5 + Se_mean6[3]*N6)/(N1 + N3 + N5 + N6);

Sensitivity_GS = (Se_mean1[4]*N1 + Se_mean2[3]*N2 + Se_mean6[4]*N6 + Se_mean7[3]*N7)/(N1 + N2 + N6 + N7);

Specificity_SW1500 = (Sp_mean1[1]*N1 + Sp_mean2[1]*N2 + Sp_mean5[1]*N5 + Sp_mean8[1]*N8)/(N1 + N2 + N5 + N8);

Specificity_SW200 = (Sp_mean3[1]*N3 + Sp_mean4[1]*N4 + Sp_mean6[1]*N6 + Sp_mean7[1]*N7)/(N3 + N4 + N6 + N7);

Specificity_BS = (Sp_mean1[2]*N1 + Sp_mean2[2]*N2 + Sp_mean3[2]*N3 + Sp_mean4[2]*N4 + Sp_mean6[2]*N6 + Sp_mean7[2]*N7)/(N1 + N2 + N3 + N4 + N6 + N7);

Specificity_gills = (Sp_mean1[3]*N1 + Sp_mean3[3]*N3 + Sp_mean5[2]*N5 + Sp_mean6[3]*N6)/(N1 + N3 + N5 + N6);

Specificity_GS = (Sp_mean1[4]*N1 + Sp_mean2[3]*N2 + Sp_mean6[4]*N6 + Sp_mean7[3]*N7)/(N1 + N2 + N6 + N7);

}

", dir = "path_to_output_directory"

)

model <- cmdstan_model(stan_program_8_pop, compile=FALSE)

model$compile()

#Model fitting

fit <- model$sample(data = data,

chains=4,

iter_warmup = 10000,

iter_sampling = 2000)

#Results

summary_allpop <- fit$summary(variables = c("prev19","prev20","prev22", "Sensitivity_SW1500","Sensitivity_SW200","Sensitivity_BS","Sensitivity_gills","Sensitivity_GS", "Specificity_SW1500","Specificity_SW200","Specificity_BS","Specificity_gills","Specificity_GS"))

write.csv2(summary_allpop, file="summary_allpop.csv")

draws <- fit$draws
