# Supplementary material for: Carp edema virus surveillance in the koi trade: early detection through shipping environment sampling and longitudinal monitoring of CEV outbreaks in a wholesaler facility
Source: Vet Res. 2025 Mar 4;56:48. doi: 10.1186/s13567-025-01476-1 (PMC11881292; doi:10.1186/s13567-025-01476-1)
Supplement: Supplementary file 3 — Additional file 3. GenBank references of the sequences produced in the present work. [file 13567_2025_1476_MOESM3_ESM.docx]

# **Additional file 3: GenBank references of the sequences produced in the present work**

| Sample | | Genbank accession number |
| --- | --- | --- |
| 2019_day00_D3_bag_swab |  | PQ336947 |
| 2019_day10_resident_batch21G1_gill_swab |  | PQ336948 |
| 2019_day00_A2_shipping_water | 1/2 | PQ336949 |
|  | 2/2 | PQ336950 |
| 2019_day10_D3_gill_swab |  | PQ336951 |
| 2019_day10_D4_gill_swab |  | PQ336952 |
| 2019_day00_E1_shipping_water |  | PQ336953 |
| 2019_day00_E4_shipping_water |  | PQ336954 |
| 2019_day20_F1_gills |  | PQ336955 |
| 2019_day22_F1_gills |  | PQ336956 |
| 2019_day00_F2_shipping_water |  | PQ336957 |
| 2019_day00_F3_shipping_water |  | PQ336958 |
| 2019_day01_F4_gills |  | PQ336959 |
| 2019_day00_F6_shipping_water |  | PQ336960 |
| 2019_day00_G_shipping_water |  | PQ336961 |
| 2020_02_resident_batch_18G3_gill_swab |  | PQ336962 |
| 2020_02_resident_batch_19D1_gill_swab |  | PQ336963 |
| 2022_day00_A2_shipping_water |  | PQ336964 |
| 2019_day00_F1_shipping_water | 1/3 | PQ336965 |
|  | 2/3 | PQ336966 |
|  | 3/3 | PQ336967 |
| 2022_day00_P_shipping_water |  | PQ336968 |
| 2022_day47_P_gill_swab |  | PQ336969 |
| 2022_day00_F1+F2_shipping_water |  | PQ336970 |
| 2022_day53_F2_gills |  | PQ336971 |
| 2020_day00_F4_shipping_water |  | PQ336972 |
| 2022_day52_D2_gills |  | PQ336973 |
| 2022_day53_resident_batch_20D2_gills | 1/2 | PQ336974 |
|  | 2/2 | PQ336975 |
