# Supplementary material for: Carp edema virus surveillance in the koi trade: early detection through shipping environment sampling and longitudinal monitoring of CEV outbreaks in a wholesaler facility
Source: Vet Res. 2025 Mar 4;56:48. doi: 10.1186/s13567-025-01476-1 (PMC11881292; doi:10.1186/s13567-025-01476-1)
Supplement: Supplementary file 4 — Additional file 4. qPCR results produced in the present work. [file 13567_2025_1476_MOESM4_ESM.docx]

**Additional file 4:** **qPCR Results produced in the present work**

Additional file 4A: qPCR results for immediate post-arrival period

| **Batch ID** | **Post-import delay** (days) | **Sampling method** | **Total DNA concentration** (ng/µL) | **CEV qPCR** | | | **carp qPCR** | |
| --- | --- | --- | --- | --- | --- | --- | --- | --- |
|  |  |  |  | **Dilution** of the extract | **Ct**  (mean of duplicates) | **Genome copies** per 2 µL of extract | **Dilution** of the extract | **Ct** |
| 19-A1 | 0 | fishbag swab | 9E+00 | 1 | NA | <10 | n.d. | n.d. |
| 19-A1 | 0 | shipping water | 9E+00 | 1 | NA | 0,0E+00 | n.d. | n.d. |
| 19-A1 | 0 | shipping water pellet | 3E+01 | 1 | NA | <10 | n.d. | n.d. |
| 19-A2 | 0 | fishbag swab | 9E+00 | 1 | NA | 0,0E+00 | n.d. | n.d. |
| 19-A2 | 0 | shipping water | 8E+00 | 1 | 35 | 3,5E+01 | n.d. | n.d. |
| 19-B1 | 0 | fishbag swab | 8E+00 | 1 | NA | 0,0E+00 | n.d. | n.d. |
| 19-B1 | 0 | shipping water | 6E+00 | 1 | 37 | 1,8E+01 | n.d. | n.d. |
| 19-B2 | 0 | fishbag swab | 7E+00 | 1 | NA | 0,0E+00 | n.d. | n.d. |
| 19-B2 | 4 | gill swab | 3E+01 | 1 | NA | 0,0E+00 | 1 | 22 |
| 19-B2 | 0 | shipping water | 8E+00 | 1 | NA | 0,0E+00 | n.d. | n.d. |
| 19-B2 | 0 | shipping water pellet | 4E+01 | 1 | NA | 0,0E+00 | n.d. | n.d. |
| 19-C | 0 | fishbag swab | 1E+01 | 1 | NA | 0,0E+00 | n.d. | n.d. |
| 19-C | 4 | gill swab | n.d. | 1 | NA | 0,0E+00 | n.d. | n.d. |
| 19-C | 4 | gills | n.d. | 1 | NA | 0,0E+00 | 1 | 23 |
| 19-C | 0 | shipping water | 1E+01 | 1 | NA | 0,0E+00 | n.d. | n.d. |
| 19-C | 0 | shipping water pellet | 1E+01 | 1 | NA | 0,0E+00 | n.d. | n.d. |
| 19-D1 | 0 | fishbag swab | 7E+00 | 1 | NA | <10 | n.d. | n.d. |
| 19-D1 | 0 | shipping water | 6E+00 | 1 | NA | 0,0E+00 | n.d. | n.d. |
| 19-D1 | 0 | shipping water pellet | 2E+01 | 1 | NA | 0,0E+00 | n.d. | n.d. |
| 19-D2 | 0 | fishbag swab | 6E+00 | 1 | NA | 0,0E+00 | n.d. | n.d. |
| 19-D2 | 0 | shipping water | 2E+01 | 1 | NA | 0,0E+00 | n.d. | n.d. |
| 19-D2 | 0 | shipping water pellet | 7E+00 | 1 | NA | 0,0E+00 | n.d. | n.d. |
| 19-D3 | 0 | fishbag swab | 5E+00 | 1 | 38 | <10 | n.d. | n.d. |
| 19-D3 | 2 | gills | 4E+01 | 1 | NA | 0,0E+00 | 1 | 23 |
| 19-D3 | 0 | shipping water | 6E+00 | 1 | NA | 0,0E+00 | n.d. | n.d. |
| 19-D3 | 0 | shipping water pellet | 1E+01 | 1 | NA | 0,0E+00 | n.d. | n.d. |
| 19-D4 | 0 | fishbag swab | 5E+00 | 1 | NA | 0,0E+00 | n.d. | n.d. |
| 19-D4 | 0 | shipping water | 1E+01 | 1 | NA | 0,0E+00 | n.d. | n.d. |
| 19-E1 | 0 | fishbag swab | 7E+00 | 1 | 41 | <10 | n.d. | n.d. |
| 19-E1 | 0 | shipping water | 8E+00 | 1 | 35 | 3,8E+01 | n.d. | n.d. |
| 19-E2 | 0 | fishbag swab | 9E+00 | 1 | 41 | <10 | n.d. | n.d. |
| 19-E2 | 0 | shipping water | 8E+00 | 1 | 38 | <10 | n.d. | n.d. |
| 19-E3 | 0 | fishbag swab | 7E+00 | 1 | 41 | <10 | n.d. | n.d. |
| 19-E3 | 0 | shipping water | 1E+01 | 1 | 36 | 3,1E+01 | n.d. | n.d. |
| 19-E4 | 0 | fishbag swab | 6E+00 | 1 | 39 | <10 | n.d. | n.d. |
| 19-E4 | 0 | shipping water | 8E+00 | 1 | 35 | 3,8E+01 | n.d. | n.d. |
| 19-F1 | 0 | fishbag swab | 7E+00 | 1 | 40 | <10 | n.d. | n.d. |
| 19-F1 | 4 | gill swab | 1E+01 | 1 | 33 | 0,0E+00 | n.d. | n.d. |
| 19-F1 | 4 | gill swab | 2E+01 | 1 | NA | 0,0E+00 | 1 | 24 |
| 19-F1 | 4 | gill swab | 3E+01 | 1 | 40 | <10 | 1 | 22 |
| 19-F1 | 4 | gill swab | 9E+00 | 1 | 41 | <10 | 1 | 26 |
| 19-F1 | 4 | gill swab | n.d. | 1 | NA | 0,0E+00 | 1 | 24 |
| 19-F1 | 4 | gill swab | n.d. | 1 | NA | 0,0E+00 | 1 | 25 |
| 19-F1 | 2 | gills | 2E+01 | 1 | NA | 0,0E+00 | 1 | 31 |
| 19-F1 | 2 | gills | 5E+01 | 1 | NA | 0,0E+00 | 1 | 31 |
| 19-F1 | 4 | gills | 1E+01 | 1 | 32 | 1,9E+02 | 1 | 22 |
| 19-F1 | 0 | shipping water | 1E+01 | 1 | 31 | 4,0E+02 | n.d. | n.d. |
| 19-F2 | 0 | fishbag swab | 1E+01 | 1 | NA | 0,0E+00 | n.d. | n.d. |
| 19-F2 | 4 | gill swab | 6E+00 | 1 | 39 | <10 | n.d. | n.d. |
| 19-F2 | 4 | gill swab | 2E+01 | 1 | 40 | <10 | n.d. | n.d. |
| 19-F2 | 3 | gills | n.d. | 10 | NA | 0,0E+00 | 10 | 26 |
| 19-F2 | 3 | gills | 7E+01 | 10 | 40 | <10 | 10 | 25 |
| 19-F2 | 4 | gills | 7E+01 | 10 | NA | <10 | 10 | 25 |
| 19-F2 | 0 | shipping water | 8E+00 | 1 | 33 | 1,5E+02 | n.d. | n.d. |
| 19-F3 | 0 | fishbag swab | 6E+00 | 1 | 38 | <10 | n.d. | n.d. |
| 19-F3 | 4 | gill swab | 3E+01 | 1 | 38 | <10 | n.d. | n.d. |
| 19-F3 | 0 | shipping water | 9E+00 | 1 | 30 | 1,1E+03 | n.d. | n.d. |
| 19-F4 | 0 | fishbag swab | 9E+00 | 1 | 38 | <10 | n.d. | n.d. |
| 19-F4 | 1 | gills | 2E+02 | 1 | 26 | 6,2E+03 | 1 | 22 |
| 19-F4 | 0 | shipping water | 1E+01 | 1 | 29 | 1,7E+03 | n.d. | n.d. |
| 19-F5 | 0 | fishbag swab | 6E+00 | 1 | 39 | <10 | n.d. | n.d. |
| 19-F5 | 0 | shipping water | 8E+00 | 1 | 35 | 7,4E+01 | n.d. | n.d. |
| 19-F6 | 0 | fishbag swab | 7E+00 | 1 | 35 | 4,4E+01 | n.d. | n.d. |
| 19-F6 | 4 | gill swab | n.d. | 1 | NA | 0,0E+00 | n.d. | n.d. |
| 19-F6 | 4 | gill swab | n.d. | 1 | NA | 0,0E+00 | n.d. | n.d. |
| 19-F6 | 0 | shipping water | 9E+00 | 1 | 29 | 1,5E+03 | n.d. | n.d. |
| 19-G | 0 | fishbag swab | 6E+00 | 1 | NA | 0,0E+00 | n.d. | n.d. |
| 19-G | 0 | shipping water | 6E+00 | 1 | 39 | <10 | n.d. | n.d. |
| 19-H | 0 | fishbag swab | 1E+01 | 1 | NA | 0,0E+00 | n.d. | n.d. |
| 19-H | 0 | shipping water | 1E+01 | 1 | NA | 0,0E+00 | n.d. | n.d. |
| 19-H | 0 | shipping water pellet | 3E+01 | 1 | NA | <10 | n.d. | n.d. |
| 20-C1 | 0 | gills | 4E+03 | 100 | 37 | 1,2E+01 | 10 | 21 |
| 20-C1 | 0 | shipping water | 2E+02 | 10 | 32 | 1,5E+02 | n.d. | n.d. |
| 20-D1 | 0 | shipping water | 1E+02 | 10 | 21 | 7,4E+04 | n.d. | n.d. |
| 20-F1 | 0 | gills | 3E+03 | 100 | 38 | <10 | 10 | 23 |
| 20-F1 | 0 | shipping water | 1E+02 | 10 | 32 | 4,8E+03 | n.d. | n.d. |
| 20-F2 | 0 | shipping water | 4E+01 | 1 | 32 | 4,6E+01 | n.d. | n.d. |
| 20-F3 | 0 | gills | 4E+03 | 100 | NA | <10 | 100 | 23 |
| 20-F3 | 0 | shipping water | 7E+01 | 1 | 31 | 8,0E+01 | n.d. | n.d. |
| 20-F4 | 0 | shipping water | 7E+01 | 1 | 26 | 2,5E+03 | n.d. | n.d. |
| 20-F5 | 0 | gills | 4E+03 | 100 | NA | <10 | 100 | 21 |
| 20-F5 | 0 | shipping water | 9E+01 | 10 | 33 | 5,8E+01 | n.d. | n.d. |
| 20-H1 | 0 | shipping water | 1E+02 | 10 | 32 | 1,6E+02 | n.d. | n.d. |
| 20-H2 | 0 | gills | 4E+03 | 100 | 39 | <10 | 100 | 21 |
| 20-H2 | 0 | shipping water | 1E+02 | 10 | 30 | 3,6E+02 | n.d. | n.d. |
| 20-H3 | 0 | gills | 4E+03 | 100 | NA | <10 | 100 | 21 |
| 20-H3 | 0 | shipping water | 2E+02 | 10 | NA | <10 | n.d. | n.d. |
| 20-I1 | 0 | gills | 3E+03 | 100 | NA | 0,0E+00 | 100 | 22 |
| 20-I1 | 0 | shipping water | 2E+02 | 10 | NA | <10 | n.d. | n.d. |
| 20-I2 | 0 | gills | 3E+03 | 100 | NA | <10 | 100 | 21 |
| 20-I2 | 0 | gills | 5E+02 | 100 | NA | <10 | n.d. | n.d. |
| 20-I2 | 0 | shipping water | 2E+02 | 10 | 36 | 1,8E+01 | n.d. | n.d. |
| 20-J1 | 0 | shipping water | 1E+01 | 1 | 31 | 8,5E+01 | n.d. | n.d. |
| 20-J2 | 0 | shipping water | 1E+01 | 1 | 31 | 1,1E+02 | n.d. | n.d. |
| 20-J3 | 0 | shipping water | 1E+01 | 1 | 30 | 2,1E+02 | n.d. | n.d. |
| 20-K1 | 0 | shipping water | 2E+02 | 10 | 24 | 1,8E+04 | n.d. | n.d. |
| 20-L1 | 0 | shipping water | 8E+01 | 10 | 37 | <10 | n.d. | n.d. |
| 20-L2 | 0 | shipping water | 8E+01 | 10 | 29 | 9,6E+02 | n.d. | n.d. |
| 20-L3 | 0 | shipping water | 7E+01 | 1 | 28 | 8,3E+02 | n.d. | n.d. |
| 20-P1 | 0 | shipping water | 9E+02 | 100 | 33 | 6,7E+01 | n.d. | n.d. |
| 22-A1 | 0 | fishbag swab | 5E+00 | 1 | NA | 0,0E+00 | n.d. | n.d. |
| 22-A1 | 1 | gill swab | 1E+02 | 10 | NA | 0,0E+00 | 10 | 24 |
| 22-A1 | 0 | shipping water | 6E+00 | 1 | NA | 0,0E+00 | n.d. | n.d. |
| 22-A2 | 0 | fishbag swab | 5E+00 | 1 | NA | 0,0E+00 | n.d. | n.d. |
| 22-A2 | 1 | gill swab | 8E+01 | 10 | NA | 0,0E+00 | 10 | 24 |
| 22-A2 | 0 | shipping water | 1E+01 | 1 | 37 | <10 | n.d. | n.d. |
| 22-B1 | 0 | fishbag swab | 5E+00 | 1 | NA | 0,0E+00 | n.d. | n.d. |
| 22-B1 | 1 | gill swab | 6E+01 | 1 | NA | <10 | 10 | 25 |
| 22-B1 | 2 | gills | 4E+03 | 100 | NA | 0,0E+00 | 10 | 19 |
| 22-B1 | 0 | shipping water | 2E+00 | 1 | NA | 0,0E+00 | n.d. | n.d. |
| 22-B2 | 0 | fishbag swab | 3E+00 | 1 | NA | 0,0E+00 | n.d. | n.d. |
| 22-B2 | 1 | gill swab | 7E+01 | 1 | NA | <10 | 10 | 26 |
| 22-B2 | 0 | shipping water | 4E+00 | 1 | NA | 0,0E+00 | n.d. | n.d. |
| 22-B3 | 0 | fishbag swab | 4E+00 | 1 | NA | 0,0E+00 | n.d. | n.d. |
| 22-B3 | 1 | gill swab | 5E+01 | 1 | NA | <10 | 10 | 25 |
| 22-B3 | 0 | shipping water | 2E+00 | 1 | NA | 0,0E+00 | n.d. | n.d. |
| 22-B4 | 0 | fishbag swab | 4E+00 | 1 | NA | 0,0E+00 | n.d. | n.d. |
| 22-B4 | 1 | gill swab | 6E+01 | 1 | NA | 0,0E+00 | 10 | 25 |
| 22-B4 | 0 | shipping water | 7E+00 | 1 | 37 | <10 | n.d. | n.d. |
| 22-D1 | 0 | fishbag swab | 5E+00 | 1 | NA | 0,0E+00 | n.d. | n.d. |
| 22-D1 | 1 | gill swab | 5E+01 | 1 | NA | <10 | 10 | 25 |
| 22-D1 | 0 | shipping water | 6E+01 | 1 | NA | 0,0E+00 | n.d. | n.d. |
| 22-D2 | 0 | fishbag swab | 5E+00 | 1 | NA | 0,0E+00 | n.d. | n.d. |
| 22-D2 | 1 | gill swab | 5E+01 | 1 | NA | 0,0E+00 | 10 | 25 |
| 22-D2 | 0 | shipping water | 1E+01 | 1 | NA | 0,0E+00 | n.d. | n.d. |
| 22-F1 | 1 | gill swab | 2E+02 | 10 | NA | 0,0E+00 | 10 | 22 |
| 22-F1 | 1 | gills | 4E+03 | 100 | NA | 0,0E+00 | 50 | 20 |
| 22-F1 | 1 | gills | 4E+03 | 100 | NA | 0,0E+00 | 50 | 22 |
| 22-F2 | 0 | fishbag swab | 6E+00 | 1 | 36 | <10 | n.d. | n.d. |
| 22-F2 | 1 | gill swab | 2E+02 | 10 | NA | 0,0E+00 | 10 | 22 |
| 22-F2 | 1 | gills | 3E+03 | 100 | NA | 0,0E+00 | 50 | 21 |
| 22-F2 | 1 | gills | 4E+03 | 100 | NA | 0,0E+00 | 50 | 20 |
| 22-F2 | 0 | shipping water | 3E+01 | 1 | 33 | 2,7E+01 | n.d. | n.d. |
| 22-G | 0 | fishbag swab | 3E+00 | 1 | NA | 0,0E+00 | n.d. | n.d. |
| 22-G | 1 | gill swab | 1E+02 | 10 | NA | <10 | 10 | 23 |
| 22-G | 0 | shipping water | 5E+01 | 1 | NA | 0,0E+00 | n.d. | n.d. |
| 22-H1 | 0 | fishbag swab | 5E+00 | 1 | NA | <10 | n.d. | n.d. |
| 22-H1 | 1 | gill swab | 1E+02 | 10 | NA | <10 | 10 | 23 |
| 22-H1 | 0 | shipping water | 2E+01 | 1 | 35 | 2,3E+01 | n.d. | n.d. |
| 22-H2 | 0 | fishbag swab | 6E+00 | 1 | NA | <10 | n.d. | n.d. |
| 22-H2 | 1 | gill swab | 8E+01 | 1 | NA | <10 | 10 | 24 |
| 22-H2 | 0 | shipping water | 8E+00 | 1 | 35 | 2,6E+01 | n.d. | n.d. |
| 22-I | 0 | fishbag swab | 5E+00 | 1 | NA | 0,0E+00 | n.d. | n.d. |
| 22-I | 1 | gill swab | 5E+01 | 1 | NA | 0,0E+00 | 10 | 25 |
| 22-I | 0 | shipping water | 4E+01 | 1 | NA | 0,0E+00 | n.d. | n.d. |
| 22-J1 | 0 | fishbag swab | 5E+00 | 1 | NA | <10 | n.d. | n.d. |
| 22-J1 | 1 | gill swab | 3E+01 | 1 | 42 | <10 | 10 | 27 |
| 22-J1 | 0 | shipping water | 1E+01 | 1 | 38 | <10 | n.d. | n.d. |
| 22-J2 | 0 | fishbag swab | 4E+00 | 1 | NA | 0,0E+00 | n.d. | n.d. |
| 22-J2 | 1 | gill swab | 5E+01 | 1 | NA | <10 | 10 | 27 |
| 22-J2 | 0 | shipping water | 3E+01 | 1 | 37 | <10 | n.d. | n.d. |
| 22-M | 0 | fishbag swab | 3E+00 | 1 | 33 | 4,0E+01 | n.d. | n.d. |
| 22-M | 1 | gill swab | 5E+01 | 1 | NA | <10 | 10 | 25 |
| 22-M | 0 | shipping water | 5E+00 | 1 | 31 | 3,7E+02 | n.d. | n.d. |
| 22-N1 | 0 | fishbag swab | 4E+00 | 1 | 37 | <10 | n.d. | n.d. |
| 22-N1 | 1 | gill swab | 6E+01 | 1 | NA | 0,0E+00 | 10 | 25 |
| 22-N1 | 0 | shipping water | 7E+00 | 1 | 34 | 3,7E+01 | n.d. | n.d. |
| 22-N2 | 0 | fishbag swab | 4E+00 | 1 | 34 | 3,0E+01 | n.d. | n.d. |
| 22-N2 | 1 | gill swab | 5E+01 | 1 | NA | <10 | 10 | 25 |
| 22-N2 | 0 | shipping water | 9E+00 | 1 | 31 | 3,3E+02 | n.d. | n.d. |
| 22-N3 | 0 | fishbag swab | 8E+00 | 1 | 31 | 1,5E+02 | n.d. | n.d. |
| 22-N3 | 1 | gill swab | 6E+01 | 1 | NA | <10 | 10 | 25 |
| 22-N3 | 0 | shipping water | 2E+01 | 1 | 30 | 4,0E+02 | n.d. | n.d. |
| 22-O1 | 0 | fishbag swab | 7E+00 | 1 | NA | <10 | n.d. | n.d. |
| 22-O1 | 1 | gill swab | 1E+02 | 10 | NA | 0,0E+00 | 10 | 23 |
| 22-O1 | 0 | shipping water | 5E+00 | 1 | 37 | <10 | n.d. | n.d. |
| 22-O2 | 0 | fishbag swab | 5E+00 | 1 | 35 | 1,1E+01 | n.d. | n.d. |
| 22-O2 | 1 | gill swab | 1E+02 | 10 | NA | 0,0E+00 | 10 | 23 |
| 22-O2 | 0 | shipping water | 8E+00 | 1 | 32 | 1,5E+02 | n.d. | n.d. |
| 22-P | 0 | fishbag swab | 3E+00 | 1 | 37 | <10 | n.d. | n.d. |
| 22-P | 1 | gill swab | 1E+02 | 10 | NA | <10 | 10 | 23 |
| 22-P | 0 | shipping water | 2E+01 | 1 | 29 | 7,3E+02 | n.d. | n.d. |

**n.d.:** not done

**NA:** No Ct value available. Sample was negative or doubtful by qPCR. If doubtful, positivity was confirmed by migrating the qPCR product on an agarose gel or performing a conventional PCR targeting *P4a* followed by Sanger sequencing, or both.

Additional file 4B: qPCR results for non-immediate post-arrival period

| Sample ID | Sampling method | Total DNA concentration (ng/µL) | CEV qPCR | | |
| --- | --- | --- | --- | --- | --- |
|  |  |  | Dilution of the extract | Ct (mean of duplicates) | Genome copies per 2 µL of DNA extract |
| 2019_day10_D4_gill_swab | gill swab | 1,1E+01 | 1 | 27 | 2,4E+03 |
| 2019_day10_OT3_gill_swab | gill swab | 1,1E+01 | 1 | 21 | 1,9E+05 |
| 2019_day10_resident_batch21G1_gill_swab | gill swab | 1,1E+01 | 1 | 30 | 3,4E+02 |
| 2019_day20_F1_gills | gills | 8,6E+01 | 1 | 25 | 2,8E+04 |
| 2019_day22_F1_gills | gills | 2,2E+01 | 1 | 34 | 6,9E+01 |
| 2020_02_resident_batch_18G3_gill_swab | gill swab | not done | 1 | 35 | 3,4E+01 |
| 2020_02_resident_batch_19D1_gill_swab | gill swab | not done | 1 | 35 | 2,2E+01 |
| 2022_day47_P_gill_swab | gill swab | 4,8E+00 | 1 | 32 | 5,4E+01 |
| 2022_day52_D2_gills | gills | 3,3E+03 | 100 | 23 | 1,6E+04 |
| 2022_day53_F2_gills | gills | 7,7E+02 | 100 | 34 | 1,8E+01 |
| 2022_day53_resident_batch20D2_gills | gills | 3,7E+03 | 100 | 32 | 6,4E+01 |
