# Supplementary material for: Carp edema virus surveillance in the koi trade: early detection through shipping environment sampling and longitudinal monitoring of CEV outbreaks in a wholesaler facility
Source: Vet Res. 2025 Mar 4;56:48. doi: 10.1186/s13567-025-01476-1 (PMC11881292; doi:10.1186/s13567-025-01476-1)
Supplement: Supplementary file 5 — Additional file 5. Phylogenetic analyses of a 412-nt fragment of the P4a gene in imported, resident koi fish batches and published sequences (n = 110). [file 13567_2025_1476_MOESM5_ESM.docx]

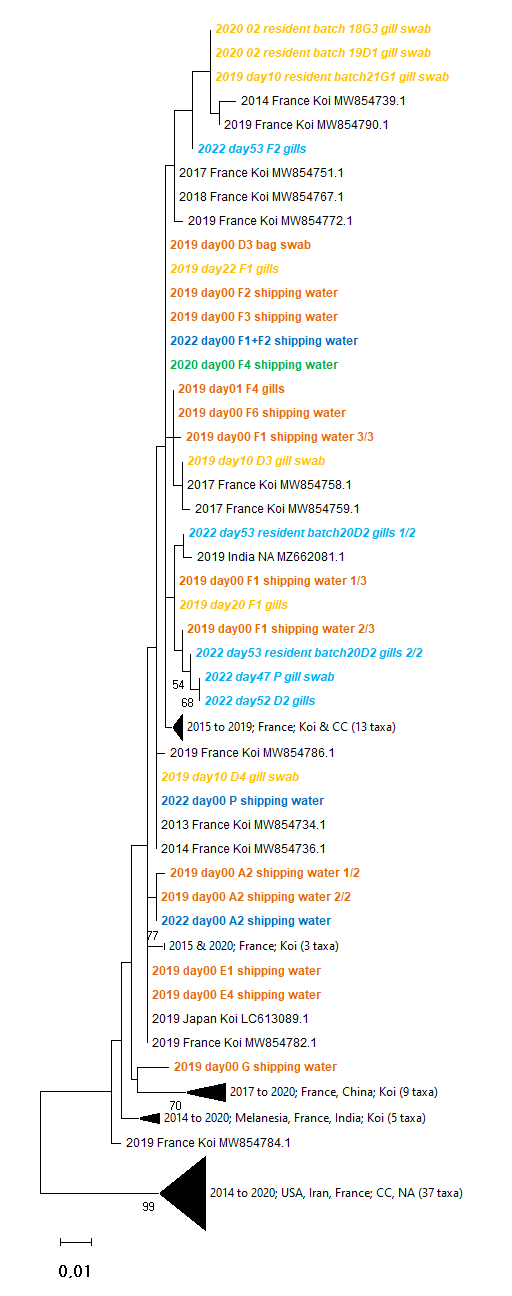


**Genogroup II**

**Genogroup I**

*The evolutionary history was inferred by using the Maximum Likelihood method and the Tamura 3-parameter model (1000 bootstraps). A discrete Gamma distribution was used to model evolutionary rate differences among sites (5 categories (+G, parameter = 0.4546)). The rate variation model allowed for some sites to be evolutionarily invariable ([+I], 70,92% sites). The percentage of trees in which the associated taxa clustered together is shown next to the branches for values higher than 50. The tree is drawn to scale, with branch lengths measured in the number of substitutions per site. In orange: 2019 importation samples; in italic yellow: samples collected between 2019 and 2020 importations; in green: 2020 importation sample; in blue: 2022 importation samples; in italic light blue: samples collected after 2022 importation. Fractions indicate different sequences coming from the same sample. CC: common carp or mirror carp; Koi: Koi carp, NA: unknown subspecies.*

*Note: The sequence originating from a CC that clusters within gII is an atypical sample, previously described by Baud* et al. *(2021).*

***Additional file 5: Phylogenetic Analyses of a 412-nt Fragment of the* P4a *Gene in Imported, Resident Koi Fish Batches and Published Sequences (n=110).***
